# Bassoon Speeds Vesicle Reloading at a Central Excitatory Synapse

**DOI:** 10.1016/j.neuron.2010.10.026

**Published:** 2010-11-18

**Authors:** Stefan Hallermann, Anna Fejtova, Hartmut Schmidt, Annika Weyhersmüller, R. Angus Silver, Eckart D. Gundelfinger, Jens Eilers

**Affiliations:** 1 Carl Ludwig Institute of Physiology, Medical Faculty, University of Leipzig, Liebigstrasse 27, 04103 Leipzig, Germany; 2 Department of Neurochemistry and Molecular Biology, Leibniz Institute for Neurobiology, 39118 Magdeburg, Germany; 3 Department of Neuroscience, Physiology and Pharmacology, University College London, Gower Street, London WC1E 6BT, UK

## Abstract

Sustained rate-coded signals encode many types of sensory modalities. Some sensory synapses possess specialized ribbon structures, which tether vesicles, to enable high-frequency signaling. However, central synapses lack these structures, yet some can maintain signaling over a wide bandwidth. To analyze the underlying molecular mechanisms, we investigated the function of the active zone core component Bassoon in cerebellar mossy fiber to granule cell synapses. We show that short-term synaptic depression is enhanced in Bassoon knockout mice during sustained high-frequency trains but basal synaptic transmission is unaffected. Fluctuation and quantal analysis as well as quantification with constrained short-term plasticity models revealed that the vesicle reloading rate was halved in the absence of Bassoon. Thus, our data show that the cytomatrix protein Bassoon speeds the reloading of vesicles to release sites at a central excitatory synapse.

## Highlights

► Generation of a transgenic mouse line that lacks Bassoon ► Normal basal transmission at mossy fiber to granule cell synapses in Bassoon mutants ► Enhanced synaptic depression within milliseconds during high-frequency transmission ► Halved rate of vesicle reloading at active zones in Bassoon knockout mice

## Introduction

Many sensory systems, such as the vestibular (Arenz et al., 2008; Bagnall et al., 2008), proprioceptive (van Kan et al., 1993), somatosensory (Jörntell and Ekerot, 2006), auditory (Lorteije et al., 2009), and visual (Azouz et al., 1997) systems, exploit a broad bandwidth of action potential frequencies to represent information as sustained rate codes. Synapses in sensory organs typically employ large, vesicle-tethering, electron-dense cytomatrix structures at their active zones (AZs), the sites where vesicles dock and fuse to release their neurotransmitter content into the synaptic cleft (Südhof, 2004). These electron-dense structures are decorated with vesicles and vary in size and shape in a species- and cell type-specific manner (Zhai and Bellen, 2004). Some extend vertically into the cytoplasm and are referred to as ribbons (Lenzi and von Gersdorff, 2001). These cytomatrix structures are thought to be critical for rapid and sustained vesicle supply at these specialized synapses, which transmit graded signals (Khimich et al., 2005; von Gersdorff et al., 1998). In contrast, central rate-coded synapses have less prominent cytomatrix structures, but some can nevertheless maintain signaling over a wide bandwidth of action potential frequencies with a relatively small number of conventional release sites (Saviane and Silver, 2006). This is achieved by a large pool of vesicles and rapid vesicle reloading to the AZ (Saviane and Silver, 2006), but the molecular mechanisms underlying this rapid reloading are unknown.

To date, at least five protein families have been characterized whose members are highly enriched at the cytomatrix of the AZs: Munc13s, RIMs, ELKS/CAST proteins, Piccolo and Bassoon, and the liprins-α (Kaeser et al., 2009; Schoch and Gundelfinger, 2006). Bassoon is a very large coiled-coil protein of ∼4000 amino acids (∼400 kDa) and is one of the core components of the cytomatrix at the AZ of both excitatory and inhibitory synapses (tom Dieck et al., 1998; Wang et al., 2009). Interestingly, whereas other AZ proteins (e.g., RIMs) are present in both vertebrates and invertebrates (e.g., *C. elegans* and *Drosophila)*, homologs of Bassoon and Piccolo (also named Aczonin; Wang et al., 2009) appear to be de novo developments of vertebrates (Altrock et al., 2003). At ribbon-type synapses, deletion of Exons 4 and 5 of the Bassoon gene leads to disrupted assembly of the cytomatrix at the AZ (Dick et al., 2003) as well as impaired auditory signaling (Buran et al., 2010; Khimich et al., 2005). At conventional synapses Bassoon is involved in trafficking and synaptic delivery of AZ material (Fejtova et al., 2009) and in partially silencing synapses (Altrock et al., 2003). However, the function of Bassoon in synaptic transmission remains unclear.

We investigated the role of Bassoon by comparing the properties of transmission at cerebellar mossy fiber to granule cell (MF-GC) synaptic connections in control and Bassoon null mutant (*Bsn^−/−^*) mice. These glutamatergic synapses appear ideally suited to investigate the mechanisms of vesicle reloading because they show rapid vesicle reloading at a limited number of release sites (Saviane and Silver, 2006). In addition, MF-GC synapses are characterized by highly synchronized vesicular release (Sargent et al., 2005), a large pool of releasable vesicles (Saviane and Silver, 2006), and firing frequencies of more than 700 Hz in vivo (Rancz et al., 2007). The excellent voltage clamp afforded by the postsynaptic granule cell leads to excitatory postsynaptic currents (EPSCs) with rise and decay kinetics in the submillisecond range with only modest desensitization (DiGregorio et al., 2007), facilitating the analysis of high-frequency signaling.

Here, we show that spontaneous EPSCs and EPSCs evoked at low frequencies are normal at MF-GC synapses in *Bsn^−/−^* mice compared to those in control mice. However, the lack of Bassoon caused a pronounced depression during high-frequency transmission that occurred within milliseconds and a delayed recovery from depression. Analysis of the presynaptic and postsynaptic mechanisms of short-term plasticity revealed that the rate of vesicle reloading at AZs of MF-GC terminals was almost halved in *Bsn^−/−^* mutants compared with controls. Thus, our data demonstrate that the cytomatrix protein Bassoon speeds high-rate vesicle reloading at AZs of a central excitatory synapse, significantly increasing the achievable rate of transmission.

## Results

### Enhanced Synaptic Depression in Cerebellar MF-GC Synapses in *Bsn^−/−^* Mice during Sustained Synaptic Signaling

**Figure 1:**
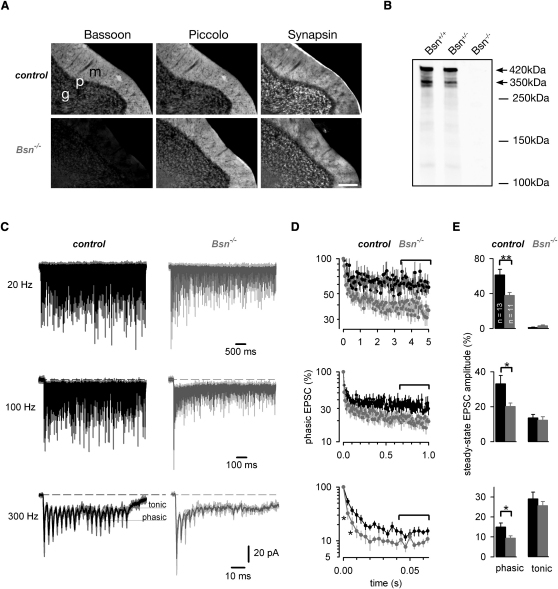
Enhanced Synaptic Depression in Cerebellar MF-GC Synapses in *Bsn^−/−^* Mice during Sustained Synaptic Signaling (A) Immunohistochemical stainings of Bassoon, Piccolo, and Synapsin in cerebellar slices of Bassoon knockout mice (*Bsn^−/−^*, bottom) and their wild-type littermates (control, top). The granular cell (g), purkinje cell (p), and molecular layer (m) are indicated (scale bar: 100 μm). (B) Western blot analysis of Bassoon expression in homogenates from whole brains of *Bsn^+/+^, Bsn^+/−^*, and *Bsn^−/−^* mice. With anti-Bassoon antibodies, two major protein bands of 420 and 350 kDa were detected in *Bsn^+/+^* and *Bsn^+/−^*, but not in *Bsn^−/−^*, mice. (C) Examples of EPSCs recorded during 20 and 100 Hz (100 stimuli) and 300 Hz (20 stimuli) in one control and one *Bsn*^−/−^ MF-GC connection. For each frequency, three traces (gray) and the corresponding average (control: black; *Bsn*^−/−^: dark gray) are superimposed. The phasic and tonic components are indicated by horizontal black lines and were automatically determined for each EPSC and each trace (see Experimental Procedures). (D) Average phasic EPSC amplitude versus time for a 20, 100, and 300 Hz stimulation for control mice (black; n = 13) and *Bsn^−/−^* mice (gray; n = 11; mean ± SEM; normalized to the first EPSC within the train; asterisks indicate significant differences, p < 0.05; note the logarithmic scale). (E) Average steady-state EPSC amplitude (see brackets in D) of phasic and tonic EPSC amplitudes in control mice (black; n = 13) and *Bsn^−/−^* mice (gray; n = 11; mean ± SEM; for data on *Bsn^ΔEx4/5^* see Figure S1).

To investigate the role of Bassoon in synaptic signaling, we developed a transgenic mouse line in which the gene encoding Bassoon was deleted (referred to as *Bsn^−/−^*). Previous studies investigating the function of Bassoon have used a Bassoon mutant (referred to as *Bsn^ΔEx4/5^*; Altrock et al., 2003; Figures S1–S3 and S6 available online), in which a 180 kDa Bassoon fragment of the Bassoon gene remained expressed. To confirm the absence of Bassoon in *Bsn^−/−^* animals, we carried out genotyping and immune labeling. Immunohistochemical staining of the cerebellum of *Bsn^−/−^* and corresponding wild-type littermates revealed normal distributions of the synaptic proteins Piccolo and Synapsin, whereas Bassoon immunoreactivity was reduced to background levels in *Bsn^−/−^* mutants (Figure 1A). Western blot analysis of the Bassoon expression in homogenates from whole brains showed two major protein bands of 420 and 350 kDa in *Bsn*^+/+^ and *Bsn*^+/−^ representing both major isoforms of Bassoon (tom Dieck et al., 1998). In their *Bsn*^−/−^ littermates, no signal was detectable, confirming that Bassoon expression was abolished in brains of mutant animals (Figure 1B).

To analyze sustained high-frequency signaling over a broad range of frequencies observed in vivo (Jörntell and Ekerot, 2006; van Kan et al., 1993), single mossy fiber inputs to cerebellar granule cells in acute brain slices were identified by the all-or-none appearance of EPSCs in response to local stimulation of graded intensity in the tissue surrounding the granule cell (Figures S1A and S1B; Silver et al., 1996). At individual MF-GC connections in *Bsn^−/−^* mice and their corresponding control littermates, EPSCs were elicited at frequencies of 20, 100, and 300 Hz with 100, 100, and 20 stimuli, respectively. At an individual connection, each frequency was evaluated at least once and in most cases three times, interleaved by >30 s intervals (Figure 1C). Unless stated otherwise, all experiments were performed on P20–28 mice at 37°C. To evaluate short-term plasticity during the trains, the phasic and tonic component of each EPSC were automatically determined for each EPSC (Figure 1C, lower left; Experimental Procedures; Saviane and Silver, 2006). The average phasic EPSC amplitudes normalized to the first amplitude in the trains were plotted against time for the frequencies investigated (Figure 1D), revealing that the degree of depression was comparable to previously published values estimated for MF-GC connections of rats at physiological temperatures (Saviane and Silver, 2006). However, comparing *Bsn^−/−^* with control revealed that the depression of the phasic EPSC amplitude was stronger in *Bsn^−/−^* (Figure 1D). In order to further quantify this effect, the degree of synaptic depression was determined for the phasic and tonic EPSCs during the steady state (brackets in Figure 1D) for each frequency at each synaptic input. The average across all connections revealed significantly smaller phasic EPSC amplitudes during steady-state while the tonic component was not significantly changed in *Bsn^−/−^* compared to that of controls (e.g., steady-state phasic EPSC for 300 Hz: 14.9% ± 2.0% and 9.4% ± 1.1%, for control and *Bsn^−/−^*, n = 13 and 11, respectively, p = 0.03; Figure 1E). Indeed, the synaptic depression of the second and third EPSC amplitude in the 300 Hz train was already significantly different (second EPSC: 55% ± 8% and 32% ± 7% for control and *Bsn^−/−^*; p < 0.05; Figure 1D). In a previous study investigating the function of Bassoon in the hippocampus of *Bsn^ΔEx4/5^* mice at 23°C, no alteration in short-term plasticity was observed (Altrock et al., 2003). We therefore repeated the experiments at cerebellar MF-GC connections in *Bsn^ΔEx4/5^* and control mice at 23°C, but found again significantly enhanced depression in *Bsn^ΔEx4/5^* (Figures S1C–S1E). These data indicate that the lack of Bassoon enhanced depression within milliseconds at MF-GC connections during sustained high-frequency signaling.

### Normal Basal MF-GC Transmission in *Bsn^−/−^*

**Figure 2:**
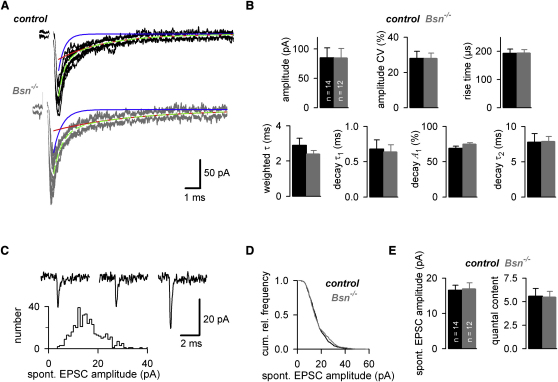
Basal MF-GC Transmission Is Normal in *Bsn^−/−^* (A) Fifteen consecutive EPSCs superimposed with the average EPSC (white) for control (top) and *Bsn^−/−^* (bottom) mice recorded at 37°C (stimulus artifacts removed). Biexponential fits to the current decay are superimposed (single components: red and blue; sum: green dashed). (B) Average EPSC amplitude, its coefficient of variation (CV), the 20%–80% rise time, the amplitude-weighted decay time constant, the decay time constant of the fast exponential component (τ_1_), its relative amplitude (*A1*), and the decay time constant of the slower exponential component (τ_2_) for control (black, n = 14) and *Bsn^−/−^* (gray, n = 12) mice. (C) Three examples of spontaneous EPSCs and the corresponding amplitude histogram of this control experiment. (D) Cumulative histogram of amplitudes of spontaneous EPSCs from all experiments of control mice (black) and *Bsn^−/−^* mice (gray, Kolmogorov-Smirnov test: p = 0.26). (E) Average amplitude of spontaneous EPSCs and estimated quantal content for control mice (black, n = 14) and *Bsn^−/−^* mice (gray, n = 12; for data on *Bsn^ΔEx4/5^* see Figure S2).

To gain insights into the mechanisms of enhanced depression in *Bsn^−/−^*, basal low-frequency transmission and its quantal components were analyzed. The amplitudes of EPSCs elicited at 1 Hz (85 ± 17 and 85 ± 16 pA, for control and *Bsn^−/−^*, n = 14 and 12, respectively, p = 0.8), their coefficients of variation (CV: 28 ± 4% and 29 ± 3%, p = 0.7, respectively), and their kinetic parameters (rise time: 192 ± 15 and 195 ± 12 μs, p = 0.7; weighted decay τ: 2.9 ± 0.4 and 2.4 ± 0.2 ms, p = 0.7, respectively) were not significantly different in control and *Bsn^−/−^* mice (Figures 2A and 2B). Comparable results were obtained at *Bsn^ΔEx4/5^* mice at 23°C (Figure S2).

The distribution of spontaneous EPSC amplitudes was slightly skewed to larger amplitudes (Figure 2C) as previously described for quantal EPSCs at this synapse (Cathala et al., 2003). A cumulative histogram of the spontaneous EPSC amplitudes from all experiments showed no statistical differences in the amplitude distribution (Figure 2D; Kolmogorov-Smirnov test: p = 0.26). The average spontaneous EPSC amplitude was similar (16.6 ± 1.4 and 17.0 ± 1.7 pA, for control and *Bsn^−/−^*, n = 14 and 12, respectively; p = 0.97; Figure 2E). In addition, the amplitude of the spontaneous EPSCs was similar to the amplitude of previously isolated miniature EPSCs (Cathala et al., 2003), consistent with the finding that spontaneous EPSCs exhibit properties of individual quantal events at this synapse (Cathala et al., 2003). Since glutamate receptors are not saturated during a quantal event (DiGregorio et al., 2007) and quanta sum linearly over a wide range of release probabilities (Sargent et al., 2005), the quantal content (i.e., the number of vesicles per EPSC) was estimated by dividing the basal evoked EPSC amplitude by the spontaneous EPSC amplitude (Del Castillo and Katz, 1954). To account for jitter in the latency of quantal release and spillover from neighboring release sites, the quantal size (*q*) was reduced by 14%, corresponding to the previously determined ratio of the stimulus- and rise-aligned quantal responses (Sargent et al., 2005). In *Bsn^−/−^*, the quantal content was similar to controls (5.6 ± 0.8 and 5.5 ± 0.6, for control and *Bsn^−/−^*, n = 14 and 12, respectively, p = 0.6; Figure 2E).

### Evoked EPSC Amplitudes Recover More Slowly from Synaptic Depression in *Bsn^−/−^* Mice Than in Control Mice

**Figure 3:**
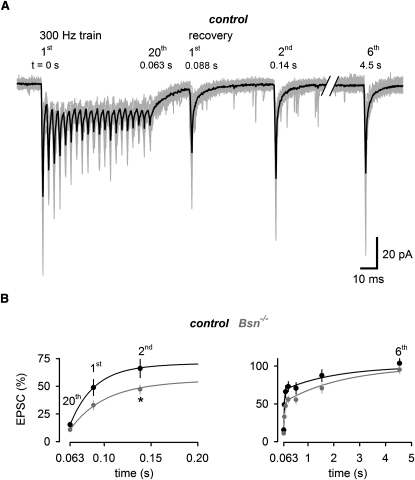
Evoked EPSC Amplitudes Recover More Slowly from Synaptic Depression in *Bsn^−/−^* Mice Than in Control Mice (A) Twenty consecutive current traces elicited by a 300 Hz train followed by test stimuli of increasing intervals (the first two and the last EPSC out of six are shown) recorded in a control mouse. The average is superimposed in black. Note the spontaneous EPSCs after the train. (B) Average phasic amplitudes of the twentieth EPSC at the end of 300 Hz trains followed by the first and second EPSC (left) and all (first–sixth) EPSCs (right) of the recovery for control mice (black; n = 13) and *Bsn^−/−^* mice (gray; n = 11; asterisks indicates significant differences) superimposed with biexponential fits (for recovery from 20 and 100 Hz trains and for data on *Bsn^ΔEx4/5^*, see Figure S3).

Next, we asked whether the enhanced depression during sustained high-frequency transmission in *Bsn^−/−^* is accompanied by alterations in the kinetics of recovery from depression. To investigate this, EPSCs were elicited by stimuli with increasing intervals following the 20, 100, and 300 Hz stimulation. In Figure 3A, 20 consecutive current traces (gray) of 300 Hz trains recorded in a control cell followed by stimuli of increasing interval (25 ms – 5 s) are superimposed with the average (black). The initial phase of the recovery after 300 Hz trains (Figure 3B, left) was slower in *Bsn^−/−^* compared with control (47% ± 4% versus 66% ± 7%, p < 0.05 at 140 ms). Biexponential fits to the recovery revealed time constants of 26 and 36 ms with amplitudes of 62% and 46% for the fast component for control and *Bsn^−/−^*, respectively, and a slower component with a time constant of ∼2 s for both control and *Bsn^−/−^* (see Figure S3E for the analysis of the recovery after 20 and 100 Hz stimulations).

### Spontaneous EPSC Amplitudes Recover Rapidly from Depression in Both *Bsn^−/−^* and Control Mice

**Figure 4:**
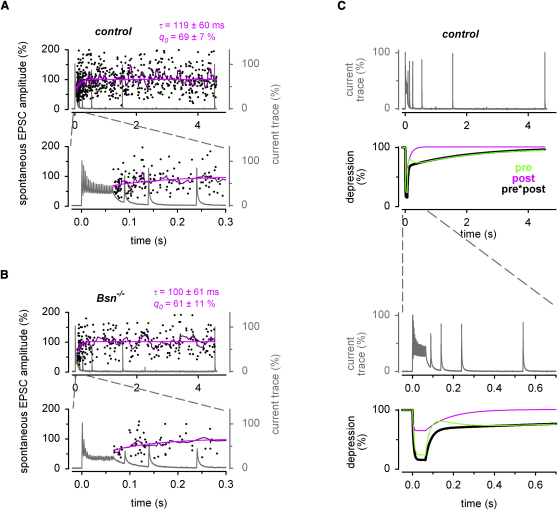
Spontaneous EPSC Amplitudes Recover Rapidly from Depression in Both *Bsn^−/−^* and Control Mice (A and B) Normalized amplitudes of spontaneous EPSCs (black) measured after the 300 Hz trains pooled across all control (A) and *Bsn^−/−^* recordings (B). A corresponding average current trace is shown in gray (note gray scale on the right). A sliding average of 11 consecutive spontaneous EPSCs amplitudes is superimposed in dark magenta. An exponential fit to the spontaneous EPSC amplitudes is superimposed in magenta (y[t] = 100 – (100 – *q_0_*) exp[−t/τ]). The initial amplitude of the spontaneous EPSC after the trains (*q_0_*) and the time constant of recovery (τ) are indicated. The 624 and 261 spontaneous EPSCs were detected in 132 and 101 300-Hz traces. Note that the average frequency of spontaneous EPSCs per MF-GC connection was not significantly different (0.4 ± 0.1 and 0.6 ± 0.2 s^−1^ for control and *Bsn^−/−^*, n = 13 and 11, respectively, p = 0.4). (C) Average current trace of a control 300 Hz experiment (gray). Below, the time course of the average depression of the phasic EPSC amplitude during and after the 300 Hz stimulations in control mice is plotted in black (monoexponential fit to the average depression and biexponential fit to the recovery). In addition, the corresponding postsynaptic (“post,” magenta) and presynaptic components of depression (“pre,” green) are shown.

To dissect presynaptic and postsynaptic components of the recovery in control and *Bsn^−/−^*, we analyzed how rapidly the postsynaptic component of the depression, which could be due to, for example, desensitization of glutamate receptors, recovers in *Bsn*^−/−^ and control mice. Therefore, we analyzed the amplitudes of spontaneous EPSCs during the recovery (Figures 4A and 4B). Exponential fits to data from all cells of the corresponding genotype revealed an initial reduction after 300 Hz trains to ∼65%, and a subsequent recovery within ∼100 ms (Figures 4A and 4B). Assuming a constant amount of glutamate per spontaneously fusing vesicle, these data indicate that *q* is decreased to ∼65% during the train and recovers with a time constant of ∼100 ms. After the 100 and 20 Hz stimulation, the average quantal size (*q_0_*) was ∼73% and ∼100%, respectively (data not shown). These estimates are comparable to previous estimates with 100 Hz experiments at MF-GC synapses in rats (Saviane and Silver, 2006). Furthermore, the kinetics of recovery in the 100 ms range is consistent with previous estimates of recovery from desensitization of glutamate receptors at MF-GC synapses in rats (DiGregorio et al., 2007; Xu-Friedman and Regehr, 2003). These data indicate that the differences in short-term plasticity between *Bsn^−/−^* and controls cannot be attributed to postsynaptic mechanisms.

From the measured postsynaptic component of short-term plasticity (i.e., *q*) during and after high-frequency transmission, the presynaptic component of short-term plasticity could be isolated by assuming that the presynaptic and postsynaptic components multiply to give the overall short-term plasticity of the phasic EPSC amplitude (Figure 4C). The presynaptic component showed a small “overshoot” directly after the 300 Hz train, which could be due to elevated intraterminal [Ca^2+^] and thus elevated release probabilities at the end of the train. Subsequently, a slow component of the presynaptic recovery from depression was apparent (green line in Figure 4C). The dissection of presynaptic and postsynaptic mechanisms of short-term plasticity, illustrated here, was important for the further mechanistic analysis.

### Analysis of Presynaptic and Postsynaptic Mechanisms Underlying Short-Term Plasticity in *Bsn^−/−^* and Control Mice

**Figure 5:**
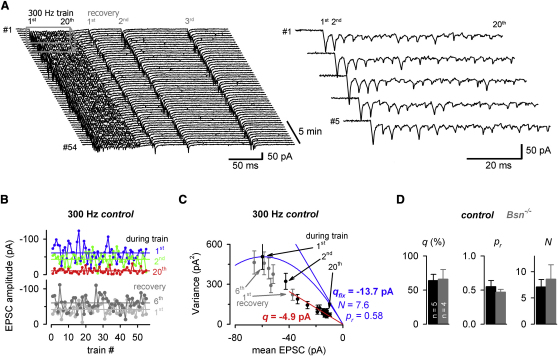
Estimating Quantal Parameters during Short-Term Plasticity with Fluctuation Analysis in *Bsn^−/−^* and Control Mice (A) Example of 54 consecutive current traces (30 s intervals) elicited by 300 Hz trains of 20 stimuli followed by test stimuli of increasing intervals (the first three out of six are shown) recorded in a control mouse. On the right, the first five traces are shown in higher resolution (cf. gray boxed area on the left; stimulus artifacts were removed for clarity). (B) Amplitude of the first (blue), second (green), and twentieth (red) EPSC during, and the first (gray) and sixth (dark gray) EPSC after, the train plotted versus the trace number for the control experiment shown in (A). The corresponding mean EPSC amplitude is indicated as a horizontal line in the corresponding color. (C) Variance of the EPSC amplitudes during the train (black) and during the recovery (gray) plotted versus the corresponding mean for the control experiment shown above, superimposed with a parabolic fit, constrained through the origin, the first EPSC during the train, and by the quantal size (*q_fix_*) determined independently from the spontaneous EPSCs measured at this connection (the blue straight line indicates the constrained initial slope *q_fix_*). The resulting number of release sites (*N*) and resting release probability (*p_r_*) are indicated. A linear fit (red) to the last 15 EPSCs during the train constrained through the origin reveals the quantal size during the train (*q*). (D) Average data for control and *Bsn^−/−^* experiments (n = 5 and 4, respectively).

To analyze the mechanisms of the enhanced synaptic depression and slower recovery in *Bsn^−/−^* mice, we first used fluctuation analysis of peak EPSC amplitudes, taking account of quantal variance and release jitter (Scheuss et al., 2002; Silver, 2003; Silver et al., 1998). Three-hundred-Hertz trains followed by test stimuli of increasing intervals were repetitively elicited at 30 s intervals (Figure 5A). The EPSC amplitudes were stable during successive trains (Figure 5B). The variance of the phasic EPSC amplitudes during and after the train was plotted versus the corresponding mean amplitude (Figure 5C). Only the variance of the first and second EPSC during the recovery was significantly lower than predicted by the blue parabolic variance versus mean relationship, which is based on a constant *q* measured independently from spontaneous EPSCs in the same cell (Figure 5C). This supported the finding that *q* recovered rapidly from depression (cf. Figure 4).

To determine the reduction in *q* during high-frequency stimulations, *q* at the end of the train was determined from the slope of a straight line through the origin fitted to the last 15 stimuli (red line; Figure 5C) and compared with *q* determined from the spontaneous EPSCs under resting conditions. The resulting reduction in *q* was 64% ± 9% and 66% ± 14% during the 300 Hz trains (n = 5 and 4, for control and *Bsn*^−/−^, respectively; p > 0.9), consistent with the estimates of desensitization from the spontaneous EPSCs. To estimate the number of functional release sites (*N*; which can also be considered as the maximum number of readily releasable vesicles at the synaptic connection) and the initial probability of vesicular release from each site (*p_r_*), the variance of the first EPSC during the train and a fixed *q* determined independently from the amplitude of the spontaneous EPSCs at that MF-GC connection (blue lines; Figure 5C) were used for the variance-mean analysis (see Experimental Procedures). The initial *p_r_* was 0.55 ± 0.09 and 0.47 ± 0.04 and *N* was 7.1 ± 1.4 and 8.6 ± 2.7 (n = 5 and 4, for control and *Bsn*^−/−^, respectively; p > 0.4). These data suggest that neither the *p_r_* nor the *N* were significantly different between *Bsn*^−/−^ and control. Furthermore, the enhanced synaptic depression in *Bsn*^−/−^ was not due to enhanced postsynaptic depression in *Bsn*^−/−^. Our data therefore suggest that the reloading of vesicles at each release site might be impaired in *Bsn*^−/−^ mice.

### Estimation of Release Properties and Vesicle Reloading at MF-GC Synapses with Short-Term Plasticity Models

**Figure 6:**
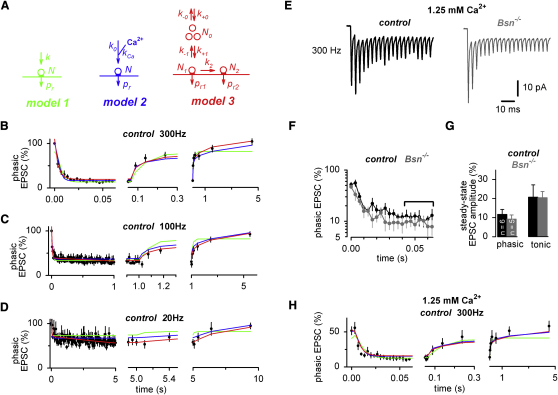
Estimating Quantal Parameters and the Kinetics of Vesicle Reloading at MF-GC Synapses with Three Short-Term Plasticity Models (A) In model 1, a MF-GC connection is characterized by *N* release sites with a release probability, *p_r_*, and a rate of vesicle reloading, *k*. In model 2, the rate of vesicle reloading, *k_0_*, is nonlinearly accelerated by the residual Ca^2+^ concentration with the maximal rate, *k_Ca_*. In model 3, a MF-GC connection is characterized by *N_1_* and *N_2_* release sites with low and high release probability, *p_r1_* and *p_r2_*, respectively. Vesicles in *N_1_* are in equilibrium with a pool of *N_0_* releasable vesicles. (B) Average phasic EPSC amplitudes of 300 Hz control experiments at 37°C superimposed with predictions of model 1, 2, and 3 in green, blue, and red, respectively. Model 1 underestimates the first EPCS amplitude and therefore does not recover to 100%. Note three different partially overlapping timescales (left: during the train, middle: end of train and beginning of recovery, right: complete 5 s lasting recovery). (C and D) Corresponding graphs for 100 and 20 Hz control trains (see Figure S4 for more details). (E) Examples of EPSCs recorded during 300 Hz stimulation (20 stimuli) in control and *Bsn*^−/−^ MF-GC connections with 1.25 mM [Ca^2+^]_e_ (average of ∼20 traces each; control: black; *Bsn*^−/−^: dark gray). (F) Average phasic EPSC amplitudes of 300 Hz trains at 1.25 mM [Ca^2+^]_e_ normalized to the basal EPSC amplitude recorded at 2 mM [Ca^2+^]_e_ in each connection for control mice (black; n = 6) and *Bsn^−/−^* mice (gray; n = 5; note the logarithmic scale). (G) Average phasic and tonic steady-state EPSC amplitudes in 300 Hz trains at 1.25 mM [Ca^2+^]_e_ for control mice (black; n = 6) and *Bsn^−/−^* mice (gray; n = 5). (H) Average phasic EPSC amplitudes of 300 Hz control experiments recorded with 1.25 mM [Ca^2+^]_e_ superimposed with the predictions of model 1, 2, and 3 in green, blue, and red, respectively, in which the release probabilities were scaled down by a factor of 2.

To quantify the kinetic properties of release and vesicular reloading, three deterministic models of short-term plasticity were used (Figure 6), taking into account the reduction in *q* during the train (cf. Figures 4 and 5). Fitting each model simultaneously to the EPSC amplitudes during and after the 300, 100, and 20 Hz data in controls gave estimates of the model parameters (see Supplemental Experimental Procedures and Hallermann et al., 2010). In the simplest possible model, a synaptic connection is characterized by a number of readily releasable vesicles (*N*) with a release probability (*p_r_*) and a rate of vesicle reloading (*k*) from an inexhaustible reserve pool (model 1; Figure 6, green). Since model 1 could not reproduce the prominent slow component of the presynaptic recovery (Figures 6B–6D), two more sophisticated plasticity models were tested that included Ca^2+^-dependent vesicle replenishment (model 2, blue; Dittman and Regehr, 1998; Hosoi et al., 2007) or two pools of readily releasable vesicles with different release probabilities and different kinetics of recovery (model 3, red; Neher, 2006; Sakaba and Neher, 2001). Indeed, such heterogeneous release probabilities would be consistent with the CV of the *p_r_* previously determined at MF-GC synapses (Sargent et al., 2005). Surprisingly, the onset and recovery time courses of the EPSC amplitudes at 20, 100, and 300 Hz were equally well described by the Ca^2+^-dependent model 2 and the Ca^2+^-independent model 3 (Figures 6B–6D; see Supplemental Discussion and Figure S4 for a detailed explanation of the models).

To further test the robustness of the models, we examined how well they predicted the onset and recovery of EPSC depression when the extracellular Ca^2+^ concentration ([Ca^2+^]_e_) was lowered to 1.25 mM (Figures 6E–6H). For each MF-GC connection, the EPSC amplitudes were normalized to the amplitude measured in 2 mM [Ca^2+^]_e_ at the beginning of the experiment. Lowering [Ca^2+^]_e_ reduced the amplitudes of the first EPSC in the train to 52% ± 6% and 47% ± 4% for control and *Bsn^−/−^* (n = 6 and 5, respectively; Figure 6F), suggesting that the initial *p_r_* was reduced by ∼50%. Note that under these conditions the depression during 300 Hz trains was not significantly different between *Bsn^−/−^* and control (steady-state phasic EPSC: 12% ± 3% and 9% ± 2%, for control and *Bsn^−/−^*, n = 6 and 5, respectively, p = 0.4; Figures 6F and 6G). After simply scaling down the initial *p_r_* by a factor of two and with all other fit parameters held constant at the best-fit parameters obtained with 2 mM [Ca^2+^]_e_, all three models predicted the time course of the 300 Hz train experiments in 1.25 mM [Ca^2+^]_e_ well (Figure 6H). Thus, without further parameter adjustment, the experiments in low [Ca^2+^]_e_ were captured well by the Ca^2+^-dependent and Ca^2+^-independent vesicle reloading models.

**Figure 7:**
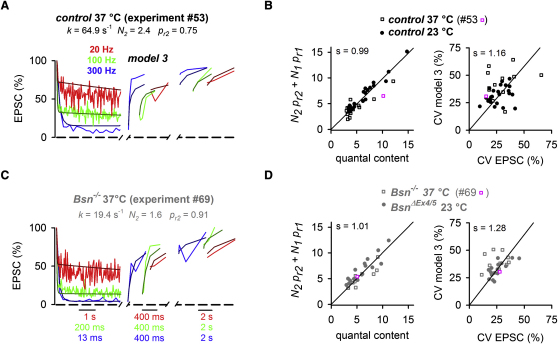
Testing the Robustness of Presynaptic Parameters Estimated with a Short-Term Plasticity Model at Individual MF-GC Connections (A) Examples of EPSC amplitudes recorded at an individual MF-GC connection in a control mouse at 37°C (20 Hz: red; 100 Hz: green; 300 Hz: blue) superimposed with the corresponding predictions of model 3 (dark colors, best-fit parameters are indicated). The recovery is shown on two different, nonoverlapping timescales and shifted horizontally. Note the color-coded timescales in (C). (B) Quantal content and coefficient of variation (CV) of EPSC amplitudes at 1 Hz stimulation (cf. Figure 1) correlated to the predictions of model 3 for each individual control connection at 37°C (open symbols) and 23°C (closed symbols), respectively (examples in A are highlighted in magenta). For calculation of CV, see Equation 6 in Supplemental Experimental Procedures. Linear fits (constrained through the origins) had slopes (s) as indicated in the graphs (nonparametric Spearman correlation coefficient, r = 0.9 and 0.3 with p < 0.0001 and p = 0.07, for quantal content and CV, respectively). (C) Corresponding example as shown in (A) for *Bsn^−/−^*. (D) Corresponding data as shown in (B) for *Bsn^−/−^* (nonparametric Spearman correlation coefficient, r = 0.9 and 0.5 with p < 0.0001 and p = 0.008, for quantal content and CV, respectively). See Figure S5 for corresponding test of model 1 and 2.

We then tested the validity of the model parameters by comparing the quantal content estimated from the models to the quantal content estimated directly from the ratio of the evoked EPSC and quantal amplitude (cf. Figure 2E) for each MF-GC connection. Figure 7A shows an example of an individual recording from a control animal. As shown in Figure 7B, there was a close to unitary relationship between the quantal content estimated from model 3 and that measured directly from the same MF-GC connections at both 23°C and 37°C. Furthermore, the predicted CV of an evoked EPSC, with the quantal parameters estimated from the fit of model 3 (which itself is deterministic, see Equation 6 in Supplemental Experimental Procedures), corresponded well to that measured directly from the same MF-GC connections at both 23°C and 37°C (Figure 7B; Del Castillo and Katz, 1954; see Figure S5 for comparable tests of model 1 and 2).

In summary, we used three release models that captured the time course of depression and recovery over a broad range of transmission frequencies and release probabilities with varying degrees of accuracy. Despite different degrees of complexity of the models, the estimated quantal parameters predict both the quantal content and the EPSC variability, indicating that they provide a reasonable estimate of *p_r_*, *N*, and *k* during and following EPSC trains across a wide range of frequencies.

### Comparison of Vesicular Reloading and Release in Control, *Bsn^−/−^*, and *Bsn^ΔEx4/5^* mice

To examine the mechanisms underlying the enhanced EPSC depression in *Bsn^−/−^* mice, we next used the models to estimate synaptic parameters from train experiments at MF-GC connections in *Bsn^−/−^* mice (for *Bsn^ΔEx4/5^*, see below). An example of such an individual experiment is superimposed with the corresponding predictions of model 3 in Figure 7C. As for the control case, the tests of robustness revealed that the synaptic parameters estimated with model 3 and the predicted CV for the *Bsn*^−/−^ mice matched well to that measured directly from the same MF-GC connections (Figure 7D; see Figure S5 for corresponding tests for model 1 and 2).

**Figure 8:**
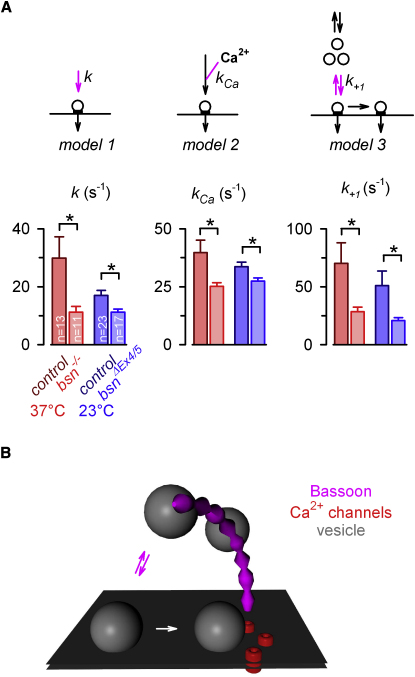
Slower Vesicle Reloading in *Bsn^−/−^* and *Bsn^ΔEx4/5^* Mice as Compared with Control Mice (A) The rates *k*, *k_Ca_*, and *k_+1_*, which are highlighted in magenta in model 1, 2, and 3, respectively, determine the speed of vesicle reloading during activity. The average reloading rates of the individual MF-GC connections are shown for the indicated temperatures and genotypes. While the remaining free parameters of the models for each MF-GC connection were on average unaltered (see Figure S6), the vesicle reloading rates were significantly reduced in *Bsn^−/−^* (37°C, red) and *Bsn^ΔEx4/5^* (23°C, blue) compared with those of corresponding control mice. (B) Illustration of the hypothesized function of Bassoon at conventional AZs. Our data show that Bassoon speeds the rapid vesicle reloading at AZs during high-frequency transmission (magenta arrows). The basal synaptic transmission and the slower component of recovery from depression, which might be limited by the maturation of vesicles closer to the Ca^2+^ channels (white arrows), appeared normal in the absence of Bassoon. Based on the comparison with electron-dense structures at ribbon-type synapses and recent high-resolution anatomical studies at conventional synapses (see Discussion), we hypothesize that the large protein Bassoon builds, anchors, or organizes the proper assembly of “miniribbons,” which speed vesicle reloading at conventional synapses.

Comparison of the presynaptic parameters determined from individual MF-GC connections of *Bsn^−/−^* and control mice revealed that *p_r_* and *N* were similar in *Bsn^−/−^* and control MF-GC connections, confirming the estimates with fluctuation analysis in a subset of experiments (cf. Figure 5). However, the rate of vesicle reloading at each release site during sustained high-frequency transmission was significantly reduced in *Bsn^−/−^* compared with control MF-GC connections, independent of the model of vesicle reloading (Figures 8A and S6). For model 3, the average rate of vesicle reloading across individual MF-GC connections was 70 ± 28 and 29 ± 4 s^−1^ in control and *Bsn^−/−^* (p = 0.02, n = 13 and 11, respectively) (right panel, Figure 8A). Fits to the average data revealed 61 and 27 s^−1^ in control and *Bsn^−/−^* mice, respectively (see Figures S6E and S6F). The consistency of the values determined from individual MF-GC connections with those from the average data further supports our approach of analyzing individual MF-GC connections to uncover significant differences and revealed that the rate of vesicle reloading in *Bsn^−/−^* MF-GC connections was halved. The number of readily releasable vesicles (*N_1_* and *N_2_*) and the release probabilities (*p_r1_* and *p_r2_*) were on average not significantly different (p > 0.3 for *N_1_* and *N_2_* and p > 0.6 for *p_r1_ and p_r2_*, Figures S6E and S6F). A significant reduction in vesicle reloading rate in *Bsn^−/−^* mice compared to control was also obtained with the two other models (Figure 8A) in which the other parameters were also unchanged (Figures S6A–S6D).

In a previous study investigating the function of Bassoon in *Bsn^ΔEx4/5^* mutants, in which a 180 kDa Bassoon fragment was still expressed (Altrock et al., 2003), no comparable alterations in short-term plasticity were found in the hippocampus. To analyze possible functions of this fragment, we repeated the experiments with *Bsn^ΔEx4/5^* mutant mice at 23°C (the temperature used by Altrock et al., 2003). However, the observed phenotype was very similar to the experiments with *Bsn^−/−^* mice at 37°C (see Figures S1–S3). Again, the mechanistic analysis revealed unaltered *p_r_* and *N* but a reduced *k* in *Bsn^ΔEx4/5^* mutant mice at 23°C compared to controls at 23°C (Figures 8A and S6). In controls at 23°C, *p_r_* and *N* were similar to controls at 37°C; however, *k* was reduced at 23°C (temperature coefficient Q_10_ = 1.2 and 1.3, for control and *Bsn^ΔEx4/5^*). These results are consistent with the findings obtained at the calyx of Held, where an increase in temperature resulted in a similar *p_r_* and vesicle pool size but accelerated vesicle recruitment (Kushmerick et al., 2006). Thus, in contrast to the finding by Altrock et al. (2003) that the absence of Bassoon partially silences hippocampal synapses, our results at physiological and room temperature indicate that Bassoon is required for efficient vesicle reloading at AZs of cerebellar MF-GC synapses.

## Discussion

In this study, electrophysiological analysis of a well-characterized conventional central excitatory synapse allowed us to isolate a specific function of the large cytomatrix protein Bassoon. The lack of Bassoon enhanced the depression of release within milliseconds during high-frequency transmission at MF-GC synapses; however, basal transmission was normal. Presynaptic and postsynaptic mechanisms of short-term plasticity were analyzed with quantal EPSC analysis, EPSC fluctuation analysis, and short-term plasticity models of vesicle release and reloading during and following high-frequency stimulation. While the release probability, the number of release sites, and the postsynaptic component of short-term plasticity were unaltered, the rate of vesicle reloading at release sites was halved in Bassoon mutants. Thus, we have identified an AZ protein that speeds vesicle reloading at a conventional synapse.

### Slow and Rapid Vesicle Recruitment at MF-GC Synapses

Our main result is that rapid reloading of vesicles at the MF-GC synapse (Saviane and Silver, 2006) depends on the presence of Bassoon, indicating that this cytomatix protein is responsible for or takes part in rapid vesicle loading. Our analysis of the recovery from high-frequency synaptic transmission also identified a second presynaptic recovery component on a much slower timescale (time constants of ∼2 s; Figures 3 and S3). Postsynaptic desensitization could not account for this component, since the amplitude of the spontaneous EPSCs recovered with a much faster time constant (∼100 ms time, Figure 4). Our models suggest that the fast and slow components of recovery in release could arise from a steep and nonlinear dependence of vesicle reloading rate on intraterminal Ca^2+^ concentration (model 2; Dittman and Regehr, 1998; Hosoi et al., 2007). However, EPSC depression during trains of different frequencies and the subsequent recovery, together with behavior at low [Ca^2+^]_e_, were equally well described by model 3, in which reloading was Ca^2+^ independent. For this model the slow recovery component was mediated by a small subpopulation of vesicles with a high *p_r_*, which recovered slowly (Neher, 2006; Sakaba and Neher, 2001). Such a nonuniform *p_r_* is consistent with the results of multiple probability fluctuation analysis at this synapse (Sargent et al., 2005). The slow recovery component was hardly affected by the lack of Bassoon (Figures 3 and S3), suggesting that Bassoon or a protein network relying on Bassoon supports the rapid reloading of vesicles that are less tightly coupled to Ca^2+^ channels and that a subsequent slower maturation step could produce tighter Ca^2+^ secretion coupling (see Figure 8B).

### Rate-Limiting Steps during High-Frequency Transmission

Although rapid release of a large number of synaptic vesicles has been described at several synapses (Hallermann et al., 2003; Hosoi et al., 2007; Saviane and Silver, 2006; von Gersdorff et al., 1998), the underlying molecular mechanisms remain debated. MF-GC synapses can signal over a wide bandwidth, which is ultimately limited by the vesicle reloading speed at a relatively small number of conventional release sites. Vesicle reloading is an integral component of the synaptic vesicle cycle that involves many steps (Neher and Sakaba, 2008; Südhof, 2004), including the clearing of the release site from the previous fusion event and the translocation, docking, and priming of the next transmitter-laden vesicle close to Ca^2+^ channels. Which of these processes are rate limiting during high-frequency transmission remains unclear (Neher and Sakaba, 2008). In the following, different possible molecular functions of Bassoon will be discussed in the light of our findings.

### Potential Molecular Functions of the Cytomatrix Protein Bassoon at AZs

Bassoon could accelerate vesicle reloading by regulating the number of vesicles clustered near the AZ to accommodate a reservoir of primed releasable vesicles, as has been proposed for the prototypical large ribbons at sensory synapses (von Gersdorff et al., 1998; Zhai and Bellen, 2004). Recent evidence obtained at the calyx of Held indicates that clearing of release sites after the fusion of a vesicle is also an important rate-limiting step in repetitive release (Hosoi et al., 2009; Kim and von Gersdorff, 2009; Neher and Sakaba, 2008; Young and Neher, 2009). Impairments of the function of the endocytotic protein Dynamin in both vertebrates and invertebrates (Hosoi et al., 2009; Kawasaki et al., 2000) lead to results similar to the ones observed in this study. This would be consistent with a role of Bassoon in Dynamin-related endocytosis. Bassoon could also support the clearance of release sites near Ca^2+^ channels by binding to, for example, RIM1 (Wang et al., 2009), which interacts with the β-subunits of voltage-gated Ca^2+^ channels (Kiyonaka et al., 2007). However, given that Bassoon has a large cytoplasmic component that could interact with intact vesicles, and that our data suggest it speeds the fast reloading and recovery of a population of low-release-probability vesicles, it seems more likely that it is involved in enhancing vesicle supply as outlined in the following.

Filamentous cytomatrix structures that link vesicles to the AZ membrane have been identified with high-resolution techniques at AZs of conventional synapses (Fernández-Busnadiego et al., 2010; Siksou et al., 2007). Furthermore, synaptic vesicles have been found to be linked to the AZ by short and long tethers (Fernández-Busnadiego et al., 2010). While short tethers are absent in samples treated with tetanus toxin, indicating that they consist of SNARE proteins, longer tethers are retained after mild synaptic stimulation, suggesting that they are involved in earlier steps of release. These long tethers might be built or anchored by the large cytomatrix protein Bassoon (see Figure 8B, Gundelfinger et al., 2003). Consistently, immunocytochemistry revealed Bassoon immuno-gold labeling at filaments emanating from the plasma membrane at the AZ (Siksou et al., 2007). Furthermore, Bassoon clusters overlapped with synaptic vesicle clusters in 3D confocal analysis of AZs at the calyx of Held (Dondzillo et al., 2010). In addition, Bassoon and Piccolo double knockout mutants showed impaired vesicle clustering at AZs (Mukherjee et al., 2010). Bassoon might directly bind to vesicles via, for example, interaction with PRA1 and Rab3 (Schoch and Gundelfinger, 2006) or anchor cytomatrix structures that bind to vesicles (Dick et al., 2003). Thus, the comparison with electron-dense structures at ribbon-type synapses suggests that the large protein Bassoon could serve as, or organize the proper assembly of, a “miniribbon” that tethers vesicles and speeds vesicle reloading at conventional synapses (Figure 8B; Verhage and Toonen, 2007).

Interestingly, the number and the size of Bassoon clusters increases during early postnatal development at the calyx of Held synapse (Dondzillo et al., 2010). Thus, immature calyces might functionally correspond to *Bsn^−/−^* mutants. In fact, detailed analysis (Taschenberger et al., 2002) indicated that mature calyces can maintain a 80%–90% higher sustained release than immature calyces, a fact only partially explained by their increased number of readily releasable vesicles (29%, consistent with 25% more ultrastructurally docked vesicles) but also by accelerated release of “undocked” vesicles (Taschenberger et al., 2002). These data correspond well to our finding of impaired release during sustained stimulation at *Bsn^−/−^* MF-GC connections. Thus, together with other filamentous AZ proteins, such as Septin 5, which overlapped with Bassoon only in immature calyces (Yang et al., 2010), the developmentally regulated accumulation of Bassoon at AZs may account for part of the functional maturation of the calyx, or at least correlate with it.

### Comparison of the Synaptic Function of Bassoon at Conventional and Ribbon-Type Synapses

The function of Bassoon has been investigated at ribbon-type synapses of cochlear inner hair cells (Buran et al., 2010; Khimich et al., 2005), which can sustain high rates of neurotransmitter release (Pangršič et al., 2010). At inner hair cell synapses of Bassoon mutant mice, synchronous vesicular release was impaired because of a reduction in the number of release sites and an impaired vesicle replenishment (Buran et al., 2010; Khimich et al., 2005; Frank et al., 2010). At MF-GC connections we found no clear indications for a reduction in the number of release sites, which suggests that the mechanisms defining the number of release sites differ in ribbon-type and conventional synapses. At conventional synapses, short-term plasticity during 10 and 50 Hz stimulation was normal at autaptic hippocampal primary cultures of *Bsn^ΔEx4/5^* mutants (Altrock et al., 2003). Furthermore, in a recent study investigating Piccolo and Bassoon double mutants (Mukherjee et al., 2010), no alterations in short-term plasticity during 10 Hz stimulation were observed at excitatory and inhibitory synapses of cultured neurons. However, a significantly enhanced depression was observed during 14 Hz stimulation at hippocampal Schaffer collateral to CA1 pyramidal cell synapses in acute brain slices of Piccolo knockout mice (Mukherjee et al., 2010). Thus, while at hippocampal synapses and synapses of cultured neurons the Bassoon phenotype of impaired vesicle reloading seems moderate or not apparent at all (Altrock et al., 2003; Mukherjee et al., 2010), a robust effect was observed at MF-GC synapses. These discrepancies may be partially due to the experimental approach (high-frequency transmission at individual synaptic connections in acute brain slices versus 10–50 Hz transmission at [autaptic] cell cultures). However, it is equally likely that the type of synapse investigated explains the discrepancies: impaired vesicle reloading would not be detected at synapses at which vesicle reloading is not rate limiting. The latter explanation is further supported by experiments with reduced [Ca^2+^]_e_. Due to the high *p_r_* of MF-GC synapses, the readily releasable pool is quickly depleted, and vesicle reloading is expected to become rate-limiting during high-frequency transmission. In experiments with lower [Ca^2+^]_e_ and thus lower *p_r_*, the phenotypic differences between *Bsn^−/−^* and control appeared smaller and were not significantly different (Figures 6E–6G). In summary, these data indicate that synapses that possess high demands on sustained release, such as cerebellar MF-GC and ribbon-type synapses, rely on the function of the cytomatrix protein Bassoon for efficient vesicle reloading.

### Implications for Information Processing

It has been reported that proprioceptive mossy fibers of the lateral reticular nucleus and the cuneocerebellar tract can fire at up to 1 kHz under appropriate peripheral activation (see references in Jörntell and Ekerot, 2006). Recently, whole-cell recordings from granule cells in vivo also indicated firing frequencies of mossy fibers at 1 kHz (Jörntell and Ekerot, 2006) and whole-cell recordings directly from mossy fibers in vivo revealed frequencies of more than 700 Hz (Rancz et al., 2007). Other cells, such as interneurons in the cat striate cortex, show comparable firing frequencies in vivo (>500 Hz; Azouz et al., 1997). Here we demonstrate that the lack of Bassoon almost halves the rate of vesicle reloading at AZs of MF-GC synapses. Our findings predict that Bassoon increases the bandwidth of rate-coded signaling and thus the fidelity of information transmission at synapses that fire at high rates. Moreover, short-term depression during high-frequency transmission enables neurons to respond to changes in firing rate rather than absolute rate (Abbott et al., 1997) and can enable inhibition-mediated neuronal gain control (Rothman et al., 2009). By reducing frequency-dependent short-term depression, Bassoon will also shift the frequency range for synaptic and neuronal gain modulation, thereby allowing multiplicative scaling of rate-coded inputs at faster timescales. Thus, the de novo development of Bassoon in invertebrates (Altrock et al., 2003) seems to be a critical component for AZs to increase their bandwidth.

In summary, the combination of genetic tools and functional characterizations of presynaptic and postsynaptic mechanisms of a specific high-fidelity synapse allowed us to link rapid vesicle reloading at a conventional synapse with the function of the AZ protein Bassoon. Our data indicate that Bassoon speeds vesicle reloading at excitatory synapses of the vertebrate brain.

## Experimental Procedures

### Mutant Animals

Mutant mice carrying a gene-trapped allele of the *Bassoon* gene were derived from Omnibank ES cell line OST486029 by Lexicon Pharmaceuticals, Inc. (The Woodlands, TX). In this line, the gene trapping of *Bassoon* was achieved by insertion of gene trapping vector VICTR 48 into Introns 1–2. Homozygous Bassoon mutant mice are here referred to as *Bsn^−/−^* and their wild-type littermates as *Bsn*^+/+^ or controls. Bassoon mutant mice lacking the central part of the protein encoded by Exons 4 and 5 of the Bassoon gene (Altrock et al., 2003) were used for the experiments at 23°C (here referred to as *Bsn^ΔEx4/5^*). The mice exhibit a mixed genetic background of C57BL/6J and 129/SvEmsJ strains, which is controlled by using sustained C57BL-backcrossed and 129 inbred mice to breed the heterozygous parents. The mice, bred at the Leibniz Institute for Neurobiology in Magdeburg, Germany, were between 20 and 28 days of age at the time of the experiments. Experiments were performed in an interleaved manner with the corresponding controls.

### Immunoblotting and Immunochemistry

Western blotting and immunohistochemical staining were performed similarly to that described previously (Altrock et al., 2003). Rabbit anti-Bassoon antibody (SAP7f; tom Dieck et al., 1998) was used for immunodetection. For details, see Supplemental Information.

### Electrophysiology

Acute cerebellar brain slices were prepared similarly to those described previously (Edwards et al., 1989). The recording temperature was either 23°C ± 1°C in experiments with *Bsn^ΔEx4/5^* mutants or 37°C ± 0.5°C (temperature controller TC-324B, Warner Instruments Corporations, Hamden, CT) in experiments with *Bsn^−/−^* mutants. See Supplemental Information for a detailed description of the electrophysiological recording conditions.

During trains of stimuli, the phasic amplitude of each EPSC of each train was detected with procedures written in Igor Pro 6.1 (Wavemetrics, Lake Oswego, OR) as the peak EPSC amplitude within a window of 1–2 ms after the stimulation artifact measured relative to a baseline (tonic component) of 0.1–0.5 ms preceding the pulse (Saviane and Silver, 2006). For each connection, the phasic and tonic steady-state amplitudes were determined as the average of the phasic and tonic amplitude of the last third of the EPCSs during the trains (indicated by the brackets in Figures 1D and 6F). For each genotype, the steady-state amplitude was subsequently averaged across all connections (Figure 1E). The EPSCs recorded here in mice were on average larger than the EPSCs recorded previously in rats (Sargent et al., 2005), but within the range of values previously obtained. Besides the species differences, a selection bias for inputs that did not exhibit failures could explain the differences. Interestingly, the normal basal EPSC amplitudes were slightly though not significantly reduced in *Bsn^ΔEx4/5^* mutant (Figure S1B) as observed in hippocampal cell cultures (Altrock et al., 2003), which could represent a dominant-negative effect of the 180 kDa Bassoon fragment remaining in *Bsn^ΔEx4/5^* mutant. Alternatively, a reduction in the basal EPSC amplitude of Bassoon mutants might have been not detected in this study at MF-GC synapses because of the mentioned bias for inputs without failures.

### Analysis of Presynaptic and Postsynaptic Mechanisms of Short-Term Plasticity

The spontaneous EPSCs were detected with a template-matching algorithm (Clements and Bekkers, 1997) applied directly after the trains, during the recovery (Figure 4). All amplitudes of spontaneous EPSCs following the corresponding trains were normalized to the spontaneous EPSC amplitude under resting conditions, plotted versus time and fitted monoexponentially. As an alternative approach, we estimated the reduction in *q* during 300 Hz trains with EPSC fluctuation analysis in a similar manner to that formerly reported (Scheuss et al., 2002; Silver, 2003; Silver et al., 1998). See Supplemental Information for a detailed description of the fluctuation analysis and of the short-term plasticity models (Hallermann et al., 2010).

### Statistical Analysis

The nonparametric Mann-Whitney-U rank sum test was used for statistical analysis unless otherwise noted. The data are reported either as mean ± SEM or as box plots with the middle line, box boundaries, and whiskers indicating the median, the 25%, 75%, 10%, and 90% quantiles, respectively. Analyses were carried out with Igor Pro 6.1 and SigmaPlot (Systat Software, Erkrath, Germany). n indicates the sample number and p denotes the significance of a Mann-Whitney-U test (^∗^p < 0.05, ^∗∗^p < 0.01).

## Supplemental Information

Document S1. Supplemental Figures, Supplemental Experimental Procedures, and Supplemental Discussion
