# Supplementary material for: Bassoon Speeds Vesicle Reloading at a Central Excitatory Synapse
Source: Neuron. 2010 Nov 18;68(4-6):710–23. doi: 10.1016/j.neuron.2010.10.026 (PMC3004039; doi:10.1016/j.neuron.2010.10.026)
Supplement: Document S1. Supplemental Figures, Supplemental Experimental Procedures, and Supplemental Discussion [file mmc1.pdf]

Neuron, volume *68*

Supplemental Data

Bassoon Speeds Vesicle Reloading at a Central Excitatory Synapse

Stefan Hallermann, Anna Fejtova, Hartmut Schmidt, Annika Weyhersmüller, R. Angus Silver, Eckart D. Gundelfinger, and Jens Eilers

I. SUPPLEMENTAL FIGURES

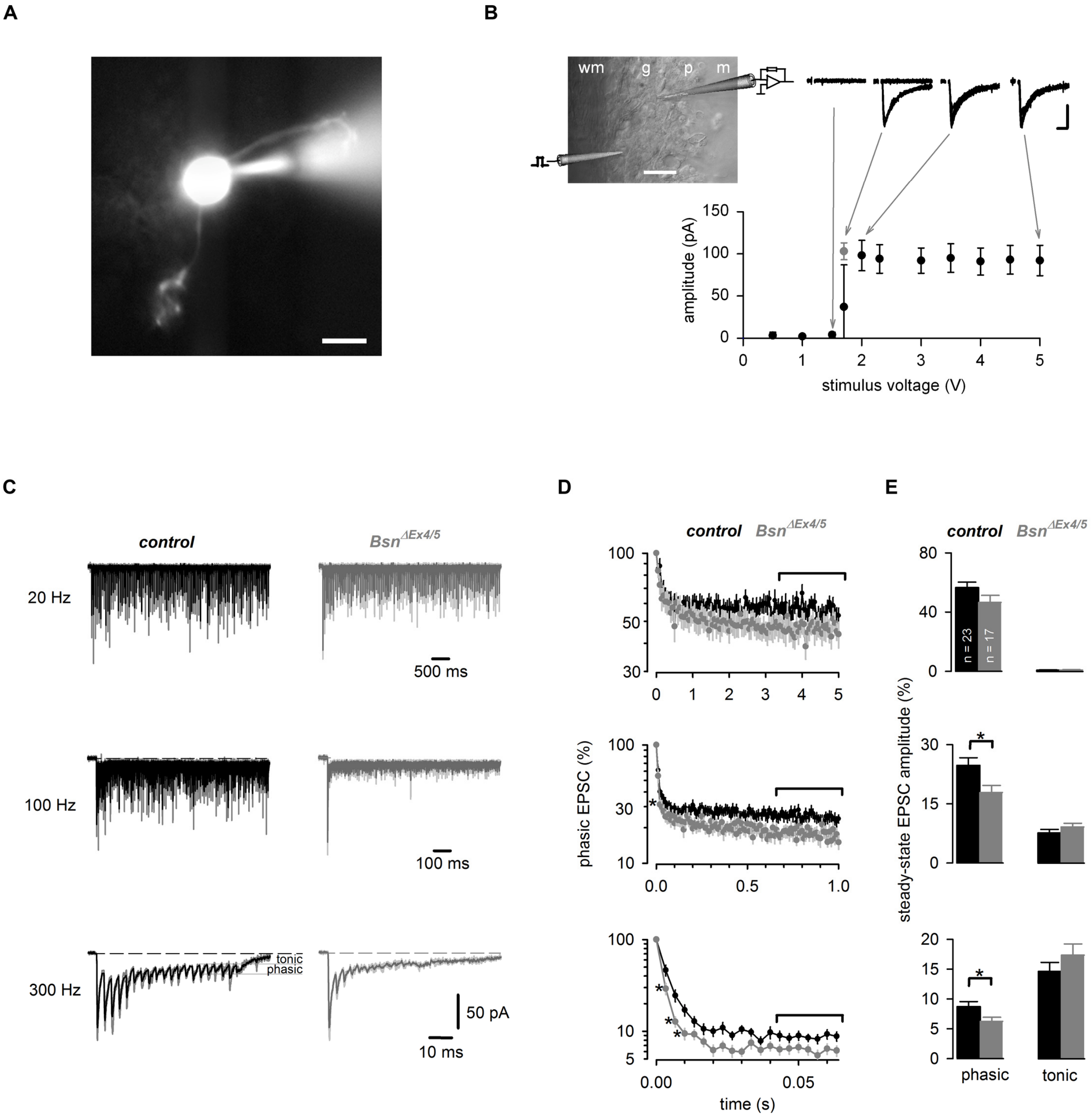

Figure S1 (related to Figure 1)

Enhanced synaptic depression in *Bsn*$^{ΔEx4/5}$ compared to control mice at 23°C during sustained synaptic signaling

(A) Fluorescence image of a whole-cell patch-clamped granule cell filled with Alexa 488 (patch pipette out of focus, on the right, scale bar 5 µm).

(B) Stimulus response curve showing the mean EPSC amplitude including (black) or excluding failures (grey) versus stimulus intensity. The small number of synapses onto the granule cell (~4) and the diffuse network of mossy fibres in the granule cell layer allowed the activation of

single mossy fibre synapses with an electrode placed in the surrounding tissue (Silver et al., 1996). Insets: Infrared differential interference contrast microscopic image of an acute cerebellar brain slice superimposed with an illustration of the pipettes. The white matter (wm) is indicated. The other layers are indicated as in panel A (scale bar 50 µm). Consecutive EPSCs elicited at the indicated stimulus intensities are shown (stimulus artefact blanked for clarity, scale bar: 50 pA, 1 ms).

(C) EPSCs recorded during 20 and 100 Hz (100 stimuli) and 300 Hz (20 stimuli). For each frequency, 3 traces (grey) and the corresponding average (control: black; $Bsn^{\Delta Ex4/5}$: dark grey) are superimposed.

(D) Average phasic EPSC amplitude versus time for a 20, 100 and 300 Hz stimulation for control (black; n = 23) and $Bsn^{\Delta Ex4/5}$ mice (grey; n = 17; normalized to the 1st EPSC within the train; error bars indicate SEM; asterisks indicate significant differences; note the logarithmic scale).

(E) The steady-state EPSC amplitude (see brackets in panel D) of phasic and tonic EPSC amplitudes for control (black; n = 23) and $Bsn^{\Delta Ex4/5}$ mice (grey; n = 17).

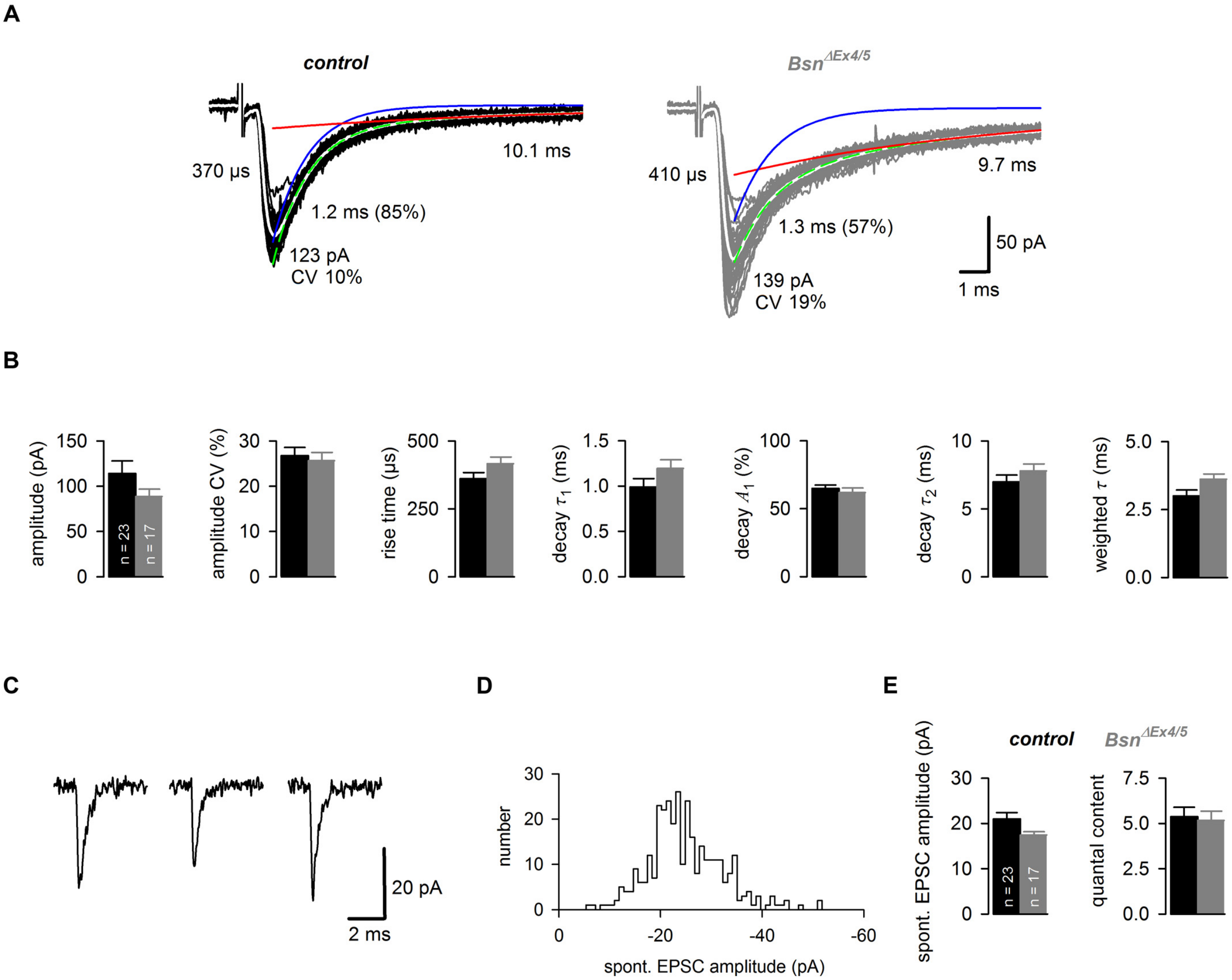

Figure S2 (related to Figure 2)

Normal basal synaptic transmission in *Bsn*$^{\Delta Ex4/5}$ and control mice at 23°C

(A) 15 consecutive EPSCs superimposed with the average EPSC (white) for control (left) and *Bsn*$^{-/-}$ mice (right) recorded at 23°C. Bi-exponential fits to the current decays are superimposed (single components: red and blue; sum: green dashed).

(B) Average EPSC amplitude, its coefficient of variation (CV), the 20-80% rise time, the decay time constant of the fast exponential component (τ_1), its relative amplitude (*A1*), the decay time constant of the slower exponential component (τ_2), and the amplitude-weighted time constant for control (black, n = 23) and *Bsn*$^{\Delta Ex4/5}$ mice (grey, n = 17). The EPSC amplitudes were 114 ± 14 and 89 ± 8 pA, n = 23 and 17, for control and *Bsn*$^{\Delta Ex4/5}$, respectively (mean ± SEM; Mann-Whitney U-test: P = 0.47; parametric t-test: P = 0.16).

(C) Three examples of spontaneous EPSCs of a control experiment.

(D) Histogram of all spontaneous EPSC amplitude of the control experiment shown in E.

(E) The average spontaneous EPSC amplitude and the estimated quantal content (EPSC / spontaneous EPCS amplitude) is shown for control (black, n = 23) and *Bsn*$^{\Delta Ex4/5}$ mice (grey, n = 17).

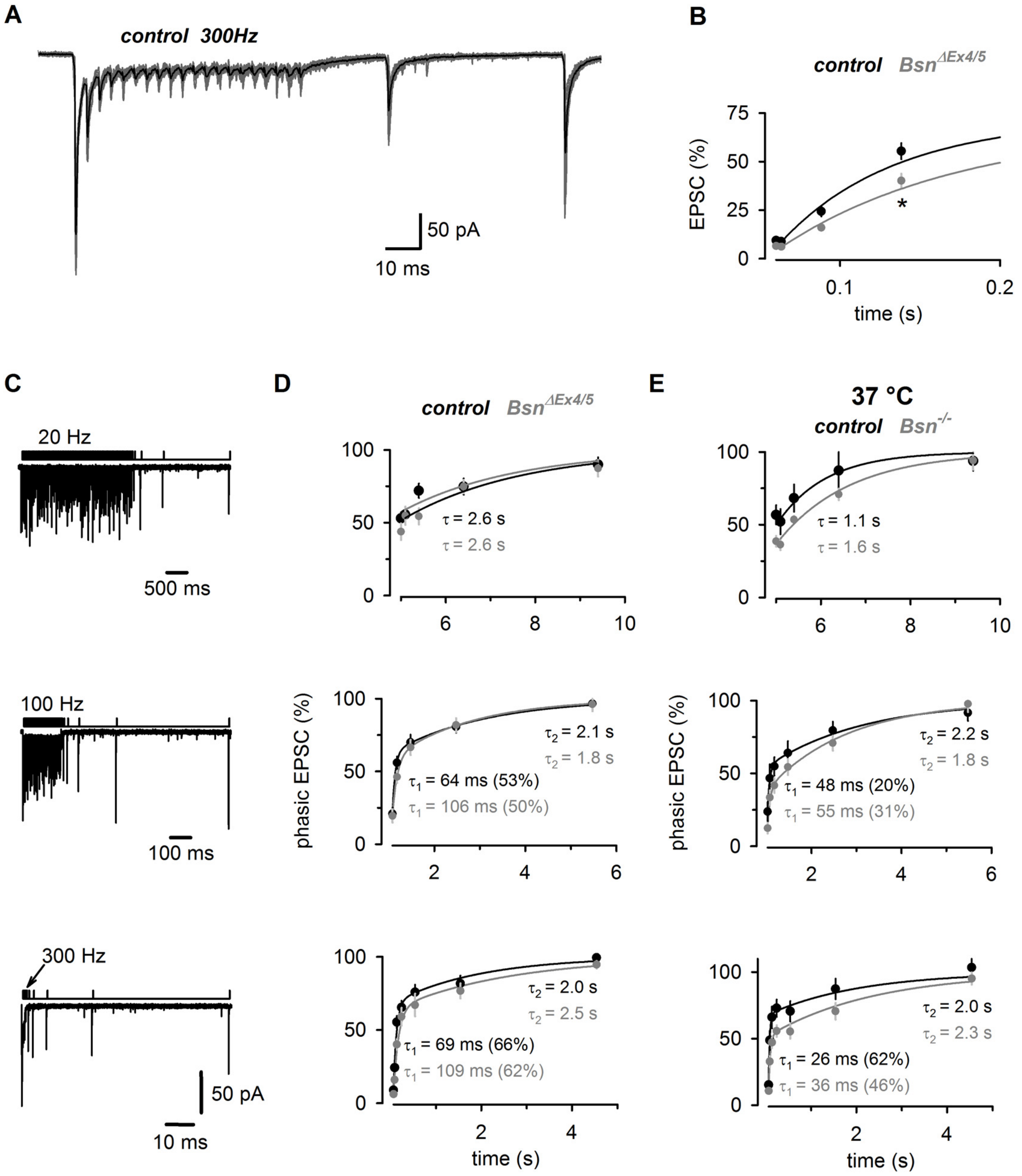

Figure S3 (related to Figure 3)

Evoked EPSC amplitudes recover more slower from synaptic depression in *Bsn*$^{\Delta Ex4/5}$ at 23°C and *Bsn*$^{-/-}$ at 37 °C than control mice

(A) 20 consecutive current traces elicited by a 300 Hz train followed by test stimuli of increasing intervals (the first 2 out of 6 are shown) recorded in a control mouse. The average is superimposed in black.

(B) Average phasic EPSC amplitude of the first two EPSCs elicited after the 300 Hz train versus time for control (black; n = 23) and *Bsn*$^{\Delta Ex4/5}$ mice (grey; n = 17; asterisks indicate significant differences). Bi-exponential fits (solid lines; constrained to reach 100 %) are superimposed.

(C) Example traces from a control mouse including EPSCs during the trains and the entire 5 s lasting recovery phase of stimuli with increasing intervals following the train. The stimulus sequence is illustrated above each trace.

(D) Average EPSC amplitudes during the recovery from 20, 100 and 300 Hz stimulation for control (black; n = 23) and *Bsn*$^{\Delta Ex4/5}$ mice (grey; n = 17). Mono- (20 Hz) and a bi-exponential fits (100 and 300 Hz) to the average time courses are shown with the corresponding time constants and amplitude proportion of the 1st component. Fits were constrained to reach 100 %.

(E) Corresponding fits to the time course of recovery measured in *Bsn*$^{-/-}$ mutants and control mice at 37°C.

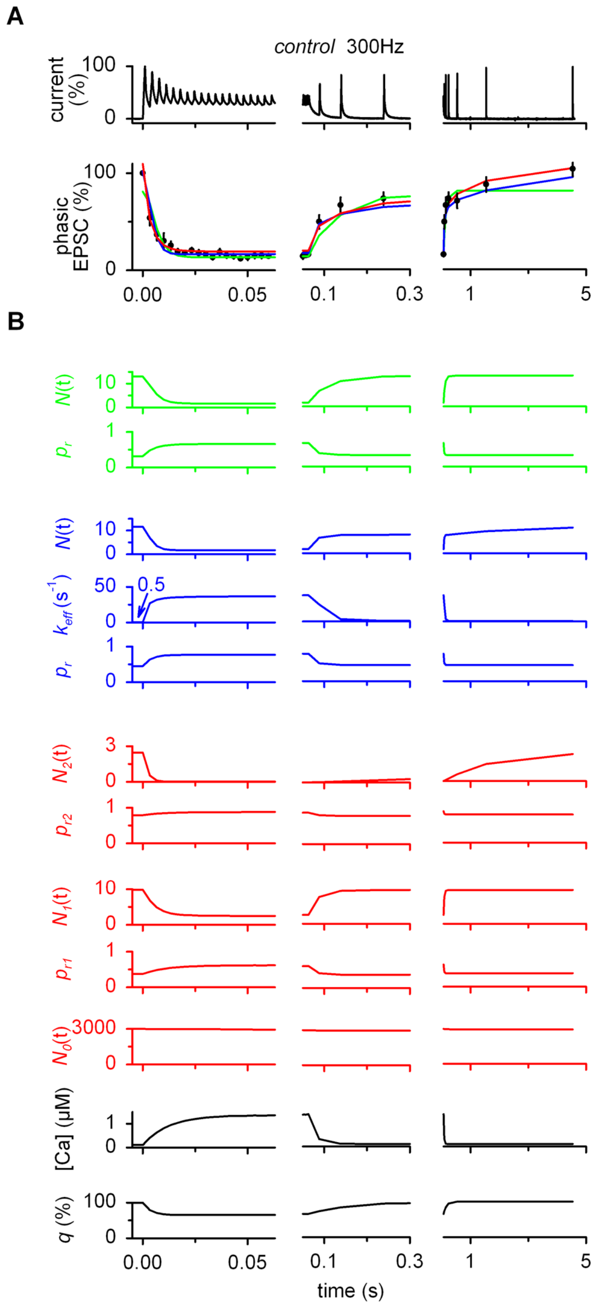

Figure S4 (related to Figure 6)

Estimating quantal parameters and the kinetics of vesicle reloading at MF-GC synapses with three short-term plasticity models

(A) As shown in panel B of Figure 6, example of a control current trace elicited by a 300 Hz train on 3 partially overlapping timescales and, below, average phasic EPSC amplitudes of all control experiments at 37 °C superimposed with predictions of model 1, 2, and 3 in green, blue, and red, respectively.

(B) Parameters of the models 1, 2, and 3 are plotted in green, blue, and red, respectively, versus time during the 300 Hz train experiments. For model 2, the resting reloading rate of

0.5 s^{-1} is indicated. Below, the assumed residual intra-terminal calcium concentration and the quantal size are plotted in black.

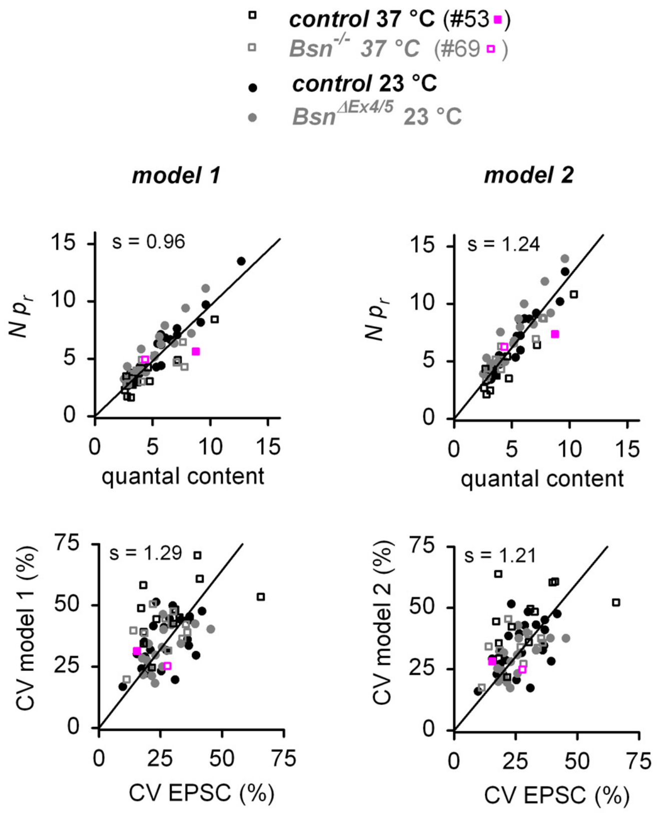

Figure S5 (related to Figure 7)

Testing the robustness of presynaptic parameters estimated with short-term plasticity model 1 and 2 at individual MF-GC connections

Quantal content (estimated as the EPSC / spontaneous EPSC amplitude) and coefficient of variation (CV) of EPSC amplitudes at 1 Hz stimulation (cf. Figure 2) correlated to the predictions of the model 1 and 2 for each individual connection at 37 °C (open symbols), 23 °C (closed symbols), control (black) and $Bsn^{-/-}$ and $Bsn^{\Delta Ex4/5}$ (grey), respectively. The two examples shown in Figure 7A and C are highlighted in pink. Linear fits (constrained to go through the origins) had slopes (s) as indicated in the graphs. The correlations were significant (non-parametric Spearman correlation coefficient, $r > 0.8$ and > 0.3 for quantal content and CV; $P < 0.05$ throughout).

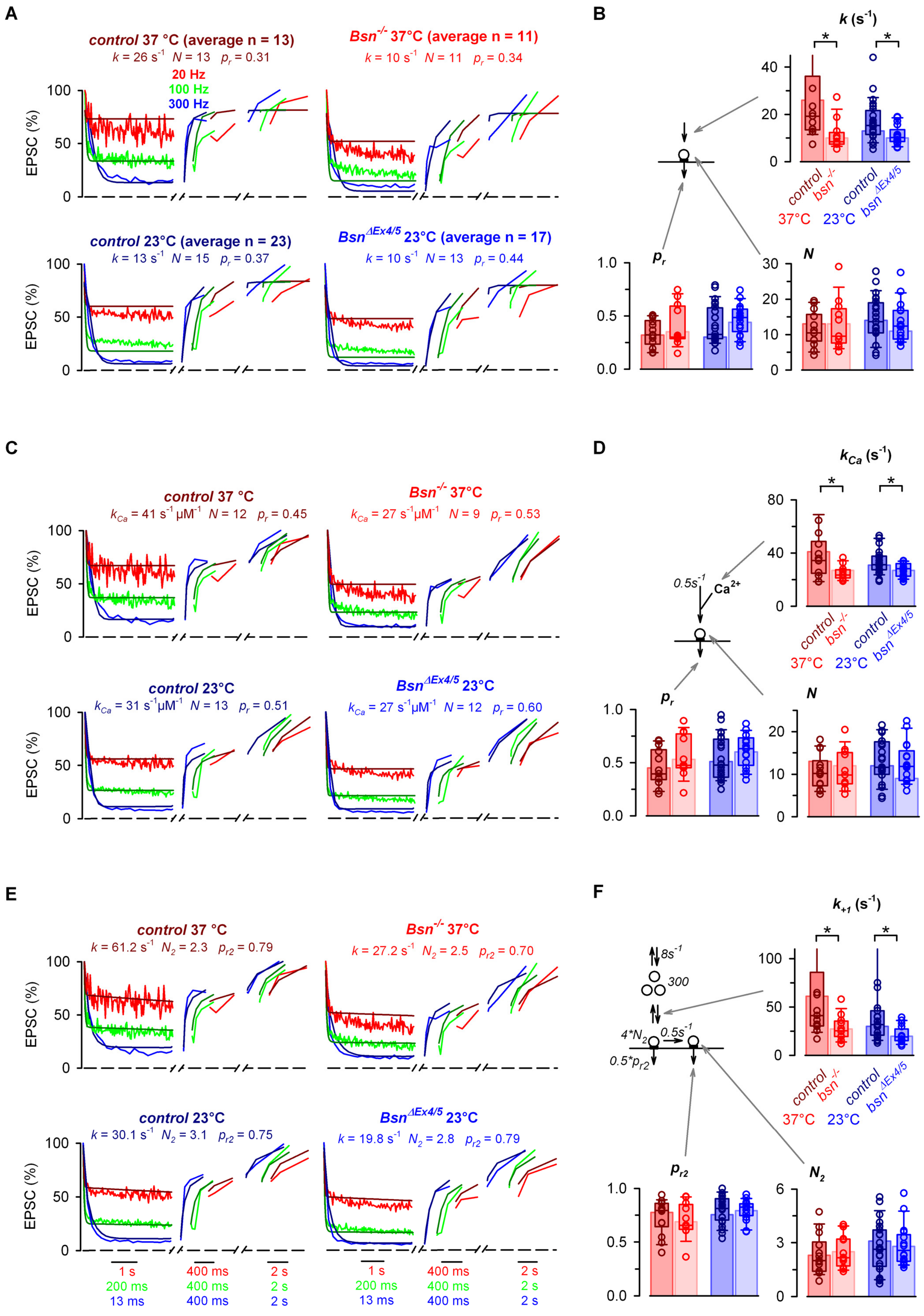

Figure S6 (related to Figure 8)

Slower vesicle reloading, normal release probability and normal number of release site with model 1 – 3 in *Bsn*$^{-/-}$ and *Bsn*$^{\Delta Ex4/5}$ compared to control mice

(A) The average time courses of synaptic short-term plasticity during 20 (red), 100 (green) and 300 Hz (blue) stimulation followed by test stimuli are superimposed with the predictions of model 1 (dark red, dark green, dark blue). To allow a direct comparison between the experimental data and the prediction of the model, the recovery is shown on two different time scales and shifted horizontally. Note the color-coded time scales in panel E. The genotype, the recording temperature, the number of experiments averaged, and the best fit parameters are indicated for each set of experiments.

(B) The parameters k, p_r, and N of model 1 estimated from each individual MF-GC connection (including 300, 100 and 20 Hz experiments) at the indicated temperatures and genotypes are shown as open circles. In addition, the parameters are displayed as box plots with the middle line, box boundaries and whiskers indicating the median, the 25, 75, 10 and 90 % quantiles, respectively. The bars represent the parameters estimated from the averages (cf. panel A). While the release probability and the number of release sites were normal, the rate of vesicle reloading was slower in *Bsn*$^{-/-}$ (37°C, red) and *Bsn*$^{\Delta Ex4/5}$ (23 °C, blue) compared with corresponding control mice.

(C and D) Corresponding data for model 2.

(E and F) Corresponding data for model 3.

II. SUPPLEMENTAL EXPERIMENTAL PROCEDURES

Immunoblotting and immunochemistry

Brain homogenates were prepared using a Teflon homogenizer (12 strokes, 900 rpm) in buffer containing 5 mM HEPES, pH 7.4, 0.32 M sucrose and Complete Proteases Inhibitor (Roche) from 2-months-old *Bsn*$^{-/-}$, *Bsn*$^{+/-}$ and *Bsn* $^{+/+}$ animals. Samples were TCA-precipitated and 10 µg of each was subjected to SDS-electrophoresis followed by Western-blotting as described previously (Altrock et al., 2003).

For immunohistochemical staining, 2-months-old *Bsn*$^{-/-}$ and *Bsn*$^{+/+}$ littermates were deeply anaesthetized using a mixture of Ketavet (Parke-Davis) and Domitor (Pfizer) and perfused transcardially with 0.9% NaCl for 1 minute followed by 4% formaldehyde in 0.1 M phosphate buffer (PB) for 10 min. Brains were removed, post-fixed in the same fixative for 20 h at 6°C, freeze-protected with 1 M sucrose in 0.1 M PB and frozen at -40°C in iso-pentane. Free-floating 25 µm thick sagittal sections were stained 48h with mouse anti-synapsin (1:2000, Synaptic Systems, Göttingen, Germany), rabbit anti-Bassoon (SAP7f, 1:2000), and guinea pig anti-Piccolo (1:2000, (Dick et al., 2001) followed by overnight incubation with Alexa 488-labeled anti-rabbit (1:2000, Invitrogen), Cy3-labeled anti-guinea pig (1:1000); Cy5-labeled anti-mouse (1:1000, both Jackson lab) antibodies. The images were taken with an upright microscope (Axioplan2; Carl Zeiss, Inc.) equipped with a CoolSNAP EZ camera (Roper Scientific, Germany) and processed with ImageJ (National Institutes of Health) and Photoshop (Adobe).

Electrophysiology

After decapitation, the vermis was removed and mounted in a chamber filled with cooled (0–2°C) artificial cerebrospinal fluid (ACSF; see below). Thick parasagittal slices (300 mm) were cut using either a HM 650V (Microm, Waldorf, Germany) or a VT1200S microtome (Leica Mikrosysteme, Wetzlar, Germany) and kept in ACSF at 37°C for 45 min and subsequently at room temperature. During the experiments, slices were perfused at 2 ml/min with ACSF containing (in mM) 125 NaCl, 2.5 KCl, 1.25 NaH_2PO_4, 26 $NaHCO_3$, 1 $MgCl_2$, 2 $CaCl_2$, 20 glucose (pH 7.4) and blockers of NMDA and GABAergic receptors (10 µM D-APV and 10 µM SR95531) and viewed using infrared differential interference contrast optics.

Whole-cell patch-clamp recordings were performed from granule cells with an EPC10/2 amplifier (HEKA Electronics, Lambrecht/Pfalz, Germany). Granule cells were identified as

previously described (capacitance of < 5 pF; (Silver et al., 1996)). Recordings were discarded if access resistance was >35 MΩ (in some recordings series resistance compensation was enabled with <90 % correction and 10 µs lag). Most granule cells were from lobe V, but there was no obvious correlation between the degree of synaptic depression and the localization of the granule cell within the cerebellum or the age of the animal. The pipette solution contained (in mM): 150 K-gluconate, 10 NaCl, 3 Mg-ATP, 0.3 Na-GTP, 10 Hepes (pH 7.3). In some experiments, 100 µM Alexa 488 was added to the patch solution and the epifluorescence signal was viewed with a CCD camera (iXon DU 897, Andor, Belfast, United Kingdom). Single mossy fibres were stimulated as described previously (Silver et al., 1996) with a patch electrode filled with ACSF placed on the surface of the surrounding tissue (Figure S1B; 0.1-10 V, duration 100 µs). In some experiments, bipolar stimulation with a theta-glass pipette was used. To facilitate finding mossy fibre inputs, in some experiments, stimulation pipettes were guided near dendritic glomeruli apparent in the fluorescence signal of Alexa 488 filled granule cells.

EPSCs were recorded at -80 mV (corrected for the liquid junction potential of 13 mV), sampled at 200 kHz (EPSCs at 1 Hz) and 20 kHz (trains of EPSCs) and filtered at 10 kHz. The amplitude and the kinetic properties of EPSCs were analyzed from the averages of about 20 EPSCs elicited at 1 Hz. The stimulus artefacts of EPCSs recorded at 37°C were removed by subtracting a double-exponential fit to the decay of the artefact current (Saviane and Silver, 2006b). The coefficient of variation (CV) was calculated as the standard deviation of the peak EPSC amplitude divided by the mean EPSC amplitude. The decay was fitted with the sum of two exponentials (baseline constrained to zero) and t = 0 was defined as the peak of the EPSC. Amplitude-weighted decay time constants, τ_w, were calculated as $(A_1\ \tau_1 + A_2\ \tau_2)/(A_1 + A_2)$, where A_1 and A_2 are amplitudes and τ_1 and τ_2 are time constants of the components.

At each investigated MF-GC connection, the size of the spontaneous EPSCs was estimated with a template matching algorithm under resting conditions (Clements and Bekkers, 1997) implemented in Neuromatic software (www.neuromatic.thinkrandom.com). Exponential fits (Figure 3 and S3) were weighted with the reciprocal of the standard deviation of the experimental errors (note that the error bars represent the standard errors in the figures). The estimated errors of the fit parameters were taken from Igor Pro and represent standard deviation.

Analysis of pre- and postsynaptic mechanisms of short-term plasticity with EPSC fluctuation analysis

In a subset of the investigated MF-GC connections, in which more than five 300 Hz trains were elicited at >30 s intervals, the variance of the phasic EPSC amplitude was calculated from sequential trains. To account for quantal jitter, the phasic EPSC amplitude was determined as the difference of the mean amplitude within an 100 µs window directly before the stimulus and at the time of the peak of the average EPSCs (Sargent et al., 2005; Saviane and Silver, 2006a). The variance of the variance was calculated according to eq. 50 in Saviane and Silver (2006a) and the error bars in Figure 5C represent the corresponding standard deviation, which were used to weight the linear fit. Based on a binomial model for synaptic transmission, the variance of the EPSC amplitude depends on the quantal size (*q;* defined as the mean quantal size at the time of the peak of the EPSC), the number of release sites (*N*), and the release probability per site (p_r), resulting in a parabolic dependence of the EPSC variance on the average EPSC amplitude. To estimate the reduction in *q* during 300 Hz trains, the relationship between the variance (σ_I^2) and the mean (*I*) of the steady state EPSCs at the end of the train (the last 15 of 20 EPSCs) were fit with the linear function:

$$\sigma_I^2 = q\,I\left(1 + CV_{QI}^2 + CV_{QII}^2\right) \qquad (1)$$

CV_{QI} and CV_{QII} are the coefficients of variation of intrasite and intersite quantal variability, respectively (Silver, 2003). CV_{QI} includes the variance arising from jitter in the quantal events at the peak of the mean EPSC. CV_{QI} and CV_{QII} were constrained to values previously obtained at MF-GC connections [0.39 and 0.31, respectively (Sargent et al., 2005)]. We here adopt the concept that the effective release probability consists of the product of the probability that a vesicle is release ready at a release site (which also can be considered as occupancy of a release site) and the release probability of such a release ready vesicle (Scheuss et al., 2002). Thus, eq. 1 is based on the assumption that during steady-state depression at the end of the train, the effective release probability per site is low because the occupancy is reduced due to depletion while p_r per occupied site (i.e. per vesicle) might still be high (as apparent in the model calculations, cf. p_{r1} and p_{r2} in Figure S4B). Next, *N* was estimated by fitting the 1st EPSC of the train in the variance-mean relationship with the function

$$\sigma_I^2 = \left[q_{fix}I - \frac{I^2}{N}\right]\left(1 + CV_{QII}^2\right) + q_{fix}I\,CV_{QI}^2 \qquad (2)$$

q_{fix} was determined independently from the mean peak amplitude of spontaneous EPSCs (*q*) recorded at the corresponding MF-GC connection (cf. Figure 2C-E) and includes a scaling

factor to account for jitter in the latency of quantal release, where $q_{fix} = q$ (15.8pA/18.4pA) corresponding to the previously determined ratio of the stimulus aligned and rise aligned quantal responses (Sargent et al., 2005). The resting p_r was calculated by dividing the mean amplitude of the 1st EPSC by $N\,q_{fix}$. To correct for contributions arising from glutamate spillover at MF-GC connections, q determined during the train was increased by 20 % and N was decreased by 28 % (Sargent et al., 2005).

Analysis of vesicle reloading at MF-GC synapses

At each investigated MF-GC connection, at least one but in most cases 3 trains of stimuli at 20, 100 and 300 Hz each (100,100, and 20 stimuli, respectively) were recorded interleaved with intervals of >30 s. Each train was followed by stimuli of increasing intervals (Magleby, 1987): 25, 50, 100, 300, 1000, 3000 ms (after 20 Hz trains the 25 and 50 ms interval were skipped).

To estimate the rate of vesicle reloading at individual MF-GC connections in control and Bassoon mutant mice, we used three established mechanistic models of vesicle recruitment to describe simultaneously the time course of the phasic EPSC amplitudes elicited during and after 20, 100, and 300 Hz stimulations (Figure 6). In model 1, a MF-GC connection was characterized by N readily releasable vesicles with a release probability (p_r) at N_{tot} release sites (assuming 100% occupancy under resting conditions). Each release site can be reloaded from an infinite pool of releasable vesicles with the rate constant, k, resulting in the following differential equation for the number of ready releasable vesicles (N):

$$\frac{d}{dt}N(t) = k\,(N_{tot} - N(t)) \qquad (3)$$

In model 2, the rate of vesicle reloading was assumed to depend on the elevation of the intra-terminal calcium concentration (Dittman and Regehr, 1998; Hosoi et al., 2007). Thus, in eq. 3, k was replaced with $\widetilde{k}$ defined according to eq. 5 of Dittman and Regehr (1998):

$$\widetilde{k}(\Delta Ca^{2+}) = k_0 + \frac{k_{Ca} - k_0}{1 + K_D / \Delta Ca^{2+}} \qquad (4)$$

where k_0 is a Ca^{2+}-independent component of reloading, k_{Ca} is the maximal rate of vesicle reloading and K_D is the residual calcium (ΔCa^{2+}) at which the Ca^{2+}-dependent component reaches its half-maximal value. A linear build up of ΔCa^{2+} was assumed, where each AP leads to a constant increase in ΔCa^{2+} by 0.4 µM (Trommershäuser et al., 2003) and decays mono-

exponentially with a time constant of 12 ms (Saviane and Silver, 2006b; see [Ca^{2+}] in Figure S4B). In a recent study, slower residual calcium transients were observed in cerebellar MF terminals of turtles (Thomsen et al., 2010), however, the transients were probably distorted by the high-affinity calcium indicator used at an ill-defined concentration. We also tested a model with linear dependence on intra-terminal calcium concentration (data not shown; Hosoi et al., 2007), however, the predicted depression during 300 Hz stimulation was too small.

Finally in model 3, a MF-GC connection is characterized by N_2 readily releasable vesicles with a high release probability (p_{r2}) and N_1 readily releasable vesicles with a lower release probability (p_{r1}). N_2 is limited by $N_{2,tot}$ release sites, which are refilled with the rate constant k_2. N_1 is defined by the equilibrium with a pool of releasable vesicles (N_0) with a refilling rate constant k_{+1} and the back-rate k_{-1} (thus, at the beginning of the simulation, t = 0, $N_1(0) = N_0(0)$ k_{+1} / k_{-1}). $N_1(0)$ might be considered as the number of functional release sites with low release probability [referred to as low p_r sites in the following; see e.g. Weis et al. (1999) for discussion of vesicle state models versus release site models]. Finally, N_0 is refilled from an infinite pool of reserve vesicles with a refilling rate constant k_{+0} and the back-rate k_{-0} (thus, $N_0(0) = k_{+0} / k_{-0}$). The number of vesicles in the three pools (N_2, N_1 and N_0) is defined by the following differential equations:

$$\frac{d}{dt}N_2(t) = k_2 N_1(t)(N_{2,tot} - N_2(t)) \qquad (5)$$
$$\frac{d}{dt}N_1(t) = k_{+1}N_0(t) - k_{-1}N_1(t) - k_2 N_1(t)(N_{2,tot} - N_2(t))$$
$$\frac{d}{dt}N_0(t) = k_{+0} - k_{-0}N_0(t) + k_{-1}N_1(t) - k_{+1}N_0(t)$$

In all three models, the release probability p_r changed according to short-term facilitation in an activity-dependent manner. In model 1 and 2, a simple model of short-term facilitation was implemented (Markram et al., 1998; Saviane and Silver, 2006b): starting from a resting release probability ($p_{r,rest}$), each action potential increases p_r by the amount $p_{r,rest}$ (1-p_r). p_r then decays mono-exponentially back to $p_{r,rest}$ with a time constant of 12 ms (Saviane and Silver, 2006b). To describe the release probabilities of both pools in model 3 with a single free parameter, p_{r1} and p_{r2} were defined according to a biophysical model of facilitation assuming different distances of the vesicles to the calcium channels (Trommershäuser et al., 2003). All other parameters describing the intracellular calcium dynamics were taken from Trommershäuser et al. (2003) only the exponential decay of the intracellular residual calcium after an action potential was constrained to 12 ms (Saviane and Silver, 2006b). α was used as

a single free parameter to define p_{r1} and p_{r2}, which approximately obeyed the relation $p_{r1} \approx 0.5\ p_{r2}$. Note that both models of facilitation led to similar results when implemented in model 1, 2 and 3, i.e., using either the biophysical-based facilitation (Trommershäuser et al., 2003) with model 1 and 2 (neglecting p_{r1}) or using the phenomenological facilitation (Markram et al., 1998) with model 3 (assuming $p_{r1} = 0.5\ p_{r2}$) led to similar EPSC amplitudes during and after the trains and consequently similar values of p_r, N, and k for each investigated MF-GC connection (data not shown).

For the simulation, we assumed the following changes of the quantal size (q) during short-term plasticity: (1) Based on the spontaneous EPSCs (Figure 4), we assumed a steady-state quantal size of 65, 73 and 99 % during 300, 100 and 20 Hz trains. (2) For the time course of reduction of q during the trains, an exponential onset kinetic with a time constant of $\tau = 1.1$ stimuli was assumed [see Figure 2g in Saviane and Silver (2006b)]. (3) After the train, an exponential recovery of q with a time constant of 100 ms was assumed for 300, 100 and 20 Hz experiments (Figure 4). The resulting time course of q is shown in Figure S4B for the 300 Hz train and the recovery.

We tested how strongly our conclusions depend on the exact assumptions of the quantal size during and after the train. Small spontaneous EPSCs at the end of the train might be missed with the template detection method. We therefore increased the steady state depression of q by 14 % for all frequencies, resulting in a steady-state q of 51, 59 and 85 % during 300, 100 and 20 Hz trains. The average resulting best-fit parameters of model 3 were: $k = 117 \pm 30$ and $50 \pm 12\ s^{-1}$ ($P < 0.05$), $p_{r2} = 0.86 \pm 0.06$ and 0.84 ± 0.05, $N_2 = 1.9 \pm 0.2$ and 2.3 ± 0.3, n = 13 and 11 for control and $Bsn^{-/-}$ at 37°C, respectively. In addition, no postsynaptic component was assumed. The average resulting best-fit parameters of model 3 were: $k = 38 \pm 6$ and $21 \pm 2\ s^{-1}$ ($P < 0.05$), $p_{r2} = 0.80 \pm 0.04$ and 0.75 ± 0.04, $N_2 = 1.9 \pm 0.2$ and 2.1 ± 0.2, n = 13 and 11 for control and $Bsn^{-/-}$ at 37°C, respectively. In summary, the absolute rate of vesicle reloading depends on the assumed degree of postsynaptic depression, however, under all tested conditions, the rate of vesicle reloading was significantly lower in $Bsn^{-/-}$ and $Bsn^{\Delta Ex4/5}$ compared with corresponding controls. Finally, the models were constrained to go through the first point of the trains (i.e. N or N_2 was not a free parameter but was a function of the release probability to result in a 1st EPCS amplitude which was equal to the average of the 1st EPSC amplitudes of the 20, the 100 and the 300 Hz trains). However, while the underestimation of the 1st EPSC amplitude in model 1 (see green lines in Figure 6B-D) was removed with this

constrain, the best-fit parameters of model 2 and 3 were very similar to the values without that constrain (data not shown).

Estimation of model parameters

To obtain robust parameter estimates from individual MF-GC connections, the slow component of recovery after depression was constrained to 2 s as found on average in control and Bassoon mutant mice at 23 and 37°C (see Figure S3). Thus, in model 2, k_0, which defines the slow recovery after the trains (when $\Delta Ca^{2+} \approx 0$), was constrained to 0.5 s^{-1}. Correspondingly in model 3, k_2, which defines the slow recovery after the trains in model 3, was constrained to 0.5 s^{-1} per release site N_2. For model 2, K_D was first used as a fourth free parameter for fitting the average control data. The obtained value of 168 nM was fixed in subsequent simulations to increase the robustness of the fit results. The MF synaptic contact is one of the smallest in the brain (160 nm diameter; Hamori and Somogyi, 1983) with an area threefold smaller than those at climbing and parallel fibres, which have 7–8 docked vesicles (Xu-Friedman and Regehr, 2003). Therefore, in model 3, the number of low p_r sites was constrained to the limiting case of four per high p_r site [i.e. $N_1(0) = 4\ N_{2,tot}$ and thus $k_{-1} = N_0(0)\ k_{+1}/(4\ N_{2,tot})$], which would correspond to a vesicle density twice that at climbing and parallel fibre synaptic contacts (Saviane and Silver, 2006b). Finally, in model 3, the number of releasable vesicles, $N_0(0)$, was constrained to 300 per low p_r site (Saviane and Silver, 2006b) and the refilling rate, k_{+0}, was constrained to 8 vesicles s^{-1} (Saviane and Silver, 2006b) [i.e. $N_0(0) = 300\ N_1(0)$, $k_{+0} = 8\ s^{-1}\ N_1(0)$, and thus $k_{-0} = k_{+0} / N_0(0)$].

The remaining three free parameters, $p_{r,rest}$, N_{tot}, and k in model 1, $p_{r,rest}$, N_{tot}, and k_{Ca} in model 2, and $p_{r2,rest}$, $N_{2,tot}$, and k_{+1} in model 3 were determined for each investigated MF-GC connection (see below). For simplicity, the suffixes *rest* and *tot* were omitted in the main text and Figs. 6, 7 and S6 [i.e. $p_{r,rest}$, N_{tot}, $p_{r2,rest}$, and $N_{2,tot}$ are referred to as p_r, N, p_{r2}, and N_2, respectively]. Furthermore, note that for a direct comparison of all models the rate k_{+1} is referred to as the rate per release site and not per vesicle in pool N_0 [i.e. as 300 k_{+1}].

Since in model 3, the effective rate of vesicle reloading depends on the rate per releasable vesicle (k_{+1}) and the number of releasable vesicles (N_0), we aimed to dissect whether the effective rate of vesicle reloading is reduced due to a slower rate of reloading per vesicle (k_{+1}) or due to fewer releasable vesicles [N_0; cf. reduced number of clustered vesicles found in Bassoon and Piccolo double knockout mice (Mukherjee et al., 2010)]. To this end, we

repeated the simulations without constraining the releasable vesicles per low p_r site to 300 but instead used $N_0(0)$ as a fourth free parameter. The following parameters were obtained from the average data: $N_0(0)$ = 1258 and 778 (127 and 78 per low p_r site), k_{+1} = 0.45 and 0.35 s^{-1}, p_{r2} = 0.78 and 0.72, and N_2 = 2.46 and 2.49, for control and $Bsn^{-/-}$ at 37 °C (fits to the average as in Figure S6E). Comparable parameters were obtained from the individual MF-GC connections. Although the variability in the four parameters estimated from individual MF-GC connections was too high to result in statistically significant differences between control and $Bsn^{-/-}$ (P > 0.5), the resulting effective rate of vesicle reloading per low p_r site was on average significantly different ($N_0(0)$ k_{+1} / [4 $N_{2,tot}$] = 54 ± 14 and 26 ± 4 s^{-1}, n = 13 and 11, for control and $Bsn^{-/-}$ at 37 °C, respectively; P < 0.05; cf. similar values obtained with model 3, see Figure 8A). Comparable results were obtained with $Bsn^{\Delta Ex4/5}$ and controls at 23 °C. These data thus indicate that the impaired vesicle reloading in MF-GC connections of Bassoon deficient mice might in part be due to less available vesicle for release, which might also explain the delayed depression during the trains in $Bsn^{-/-}$ and $Bsn^{\Delta Ex4/5}$ (Figure S6). However, the mathematical description of the reloading rate as the product of a rate per vesicles and the number of releasable vesicles is most likely a simplification. Vesicles near the release site are probably reloaded more rapidly compared with more remote vesicles. Thus, our finding that already the 2nd EPSC in 300 Hz trains was significantly reduced in $Bsn^{-/-}$ and $Bsn^{\Delta Ex4/5}$ (Figure 1 and S1) indicates that rapid reloading processes near the AZ (which are probably independent of the number of remote vesicles) were severely impaired in Bassoon deficient mice. Alternatively, a slight increase in the release probability, which was not detected with fluctuation analysis nor with the short-term plasticity models, could contribute to the rapid onset of depression in $Bsn^{-/-}$.

To further address the robustness of the estimated model parameters, the experimentally determined quantal content and the CV of the peak EPSC amplitude under resting conditions (Figure 2E and B) were compared with the values estimated from the models on the basis of the train experiments for each MF-GC connection (Figure 7 and S6). The CV was calculated as CV = σ/mean, where σ^2 was defined as (Silver, 2003):

$$\sigma^2 = Nq^2 p_r (1-p_r)(1+CV_{QII}^2) + Nq^2 p_r CV_{QI}^2 \qquad (6)$$

and the mean as $N\ q\ p_r$. The intrasite and intersite quantal variability CV_{QI} and CV_{QII} were constrained to values previously obtained at MF-GC connections [0.39 and 0.31, respectively (Sargent et al., 2005)]. The quantal size was calculated from the amplitudes of the spontaneous EPSCs as described above [i.e. 14% reduction (Sargent et al., 2005)]. For model

3, CV was defined as the square root of the sum of the variance of pool 1 and pool 2 divided by the mean ($N_2\ q\ p_{r2} + N_1\ q\ p_{r1}$), assuming uncorrelated linearly adding variances of pool 1 and pool 2.

Implementation of the simulations

Starting from the analytical solution of the differential equations, the number of vesicles within the pools were numerically calculated with a fifth-order Cash-Karp Runge-Kutta method with adaptive step-size control (Hallermann et al., 2010; Press et al., 2002). The change in p_r was calculated analytically (Markram et al., 1998; Saviane and Silver, 2006b) or numerically (Trommershäuser et al., 2003). The phasic EPSC amplitude was calculated by the number of released vesicles per stimulus [p_r(t) N(t) and for model 3, p_{r2}(t) N_2(t) + p_{r1}(t) N_1(t)] multiplied by the quantal size [q(t); see Figure S4B], assuming linear summation of the quanta (Sargent et al., 2005; Saviane and Silver, 2006b).

To compare the predictions of the model with the experimental data obtained for each MF-GC connection, the sum of the squared differences between the measured EPSC amplitudes and the predictions of the model were calculated. Exponential sampling was used during the train, i.e. only the following stimuli were considered for χ^2 calculations: number 1, 2, 3, 4, 5, 6, 8, 10, 13, 17, 23, 30, 40, 54, 73, 98 of the 100 EPSCs of the 20 and 100 Hz trains and number 1, 2, 3, 4, 5, 6, 8, 10, 13, 17, 18, 19, 20 of the 20 EPSCs of the 300 Hz train. All EPSCs elicited during recovery were considered for χ^2 calculations.

Simulations were implemented in C++ (XCode 3.0, Developer Tool, OS X, Apple, Cupertino, CA, USA). A simplex algorithm (Press et al., 2002) was used to optimize the free parameters for each MF-GC connection consecutively. Subsequently, the three trains of all investigated MF-GC connections were plotted with the predictions of the model superimposed using Mathematica 5.0 (Wolfram Research, Champaign, IL, USA) for visual inspection. Simulation and plotting lasted only several seconds on a conventional 2.7 GHz dual core computer, which allowed to gain a better understanding of the reliability and the constrains of the parameters. After the fit was completed, internal estimates of the errors of the best-fit parameters were calculated as the square roots of the diagonals of the covariance matrix (Colquhoun et al., 2003). To confirm that the global minimum was reached, the best-fit parameters were shown to be independent of the starting values within a plausible range.

III. SUPPLEMENTAL DISCUSSION OF THE SHORT-TERM PLASTICITY MODELS

As a starting point, we used the simplest possible model, in which a synaptic connection is characterized by a number of readily releasable vesicles (N) with a release probability (p_r) and a rate of vesicle reloading at an empty release site (k) from an inexhaustible reserve pool (model 1, Figure 6 green, see Supplemental Experimental Procedures for more details). Fitting this simple model, simultaneously, to the EPSC amplitudes during and after the 300, 100 and 20 Hz data in controls gave an estimate of ~10 N with an p_r of 0.37 and a k of 26 s^{-1}. The average phasic EPSC amplitudes are compared with these best fit predictions of model 1 in green for control mice (Figure 6B-D). During the 300 Hz train (Figure 6B), N was depleted to 25 %, but the p_r was increased to 0.61 from 0.37 according to a simple model of facilitation (Markram et al., 1998; see [Ca^{2+}] trace in Figure S4 and Supplemental Experimental Procedures). This model of facilitation in combination with the kinetics of vesicles reloading predicted the presynaptic overshoot after the high-frequency stimulations (cf. green line in Figure 4C), which is not apparent in Figure 6 because the postsynaptic component of short-term plasticity (see q trace in Figure S4) was included in the calculations. However, the model could not account for the recovery at intermediate times following 20 Hz stimulation, or the prominent slow component of the presynaptic recovery. Moreover, p_r predicted by the model was lower than estimates from fluctuation analysis both here (Figure 5D) and in previous studies (Sargent et al., 2005; Saviane and Silver, 2006b). We therefore applied a more sophisticated plasticity model that included Ca^{2+}-dependent vesicle replenishment.

In the second model (Figure 6, blue), the rate of vesicle reloading, k_0, was accelerated by residual calcium concentration in the terminal in a Michaelis–Menten-type manner (see eq. 4 in Supplemental Experimental Procedures; Dittman and Regehr, 1998). The rate k_0 was set to 0.5 s^{-1} to reproduce the slow component of the recovery (~2 s; Figure S3D and E) and K_D and k_{Ca} were used as free parameters. The data were best described with K_D = 168 nM and k_{Ca} = 41 s^{-1} (see Figure S4 and Supplemental Experimental Procedures for details on the assumed calcium dynamics; to fit individual MF-GC connections, K_D was constrained to 168 nM while k_{Ca} remained a free parameter). During the 300 Hz trains the assumed calcium reached values of ~1 µM and, thus, the rate of vesicle reloading was accelerated to ~40 s^{-1}.

Since Ca^{2+}-dependent vesicle reloading is difficult to disentangle from the Ca^{2+} dependences of release and facilitation and from postsynaptic saturation (where present), we examined whether the time courses of depression and recovery at the different frequencies could be

accounted for by a Ca^{2+}-independent vesicle reloading mechanism. To account for the steady-state depression, the slow component of the depression at 20 Hz, together with the fast and slow components of the recovery following trains, we developed a model with a pool of vesicles that could be recruited and a subsequent maturation step that increased the release probability of a fraction of the vesicles. In this third model (Figure 6, red), release at a MF-GC connection was described by N_2 ready releasable vesicles with a high release probability, p_{r2}, and N_1 release sites with a lower release probability, p_{r1}. The vesicles in N_1 are in equilibrium with a large pool of N_0 releasable vesicles [N_0; Saviane and Silver, 2006b]. Thus, releasable vesicles in pool N_0 become release competent in pool N_1 with a rate of k_{+1}. Subsequently, some vesicles can transit with a slower rate, k_2, to the pool N_2. This rate was set to 0.5 s^{-1} to reproduce the slow component of the recovery (~2 s; Figure S3D and E) and might represent a slow maturation of vesicles closer to calcium channels (Bucurenciu et al., 2008; Sakaba and Neher, 2001; Young and Neher, 2009). During fitting, k_{+1}, p_{r2} and N_2 were free parameters, while the remaining parameters in the model were constrained (see Supplemental Experimental Procedures). The average control data (Figure 6B-D) were best described by 2.3 and 10 vesicles in pool N_2 and N_1 with an initial release probability of 0.8 and 0.5, respectively. The rate of reloading (k_{+1}) was ~60 s^{-1}, consistent with previous estimates (Saviane and Silver, 2006b).

SUPPLEMENTAL REFERENCES

Altrock, W.D., tom Dieck, S., Sokolov, M., Meyer, A.C., Sigler, A., Brakebusch, C., Fassler, R., Richter, K., Boeckers, T.M., Potschka, H., *et al.* (2003). Functional inactivation of a fraction of excitatory synapses in mice deficient for the active zone protein bassoon. Neuron *37*, 787-800.

Bucurenciu, I., Kulik, A., Schwaller, B., Frotscher, M., and Jonas, P. (2008). Nanodomain coupling between Ca^{2+} channels and Ca^{2+} sensors promotes fast and efficient transmitter release at a cortical GABAergic synapse. Neuron *57*, 536-545.

Clements, J.D., and Bekkers, J.M. (1997). Detection of spontaneous synaptic events with an optimally scaled template. Biophys J *73*, 220-229.

Colquhoun, D., Hatton, C.J., and Hawkes, A.G. (2003). The quality of maximum likelihood estimates of ion channel rate constants. J Physiol *547*, 699-728.

Dick, O., Hack, I., Altrock, W.D., Garner, C.C., Gundelfinger, E.D., and Brandstätter, J.H. (2001). Localization of the presynaptic cytomatrix protein Piccolo at ribbon and conventional synapses in the rat retina: comparison with Bassoon. J Comp Neurol *439*, 224-234.

Dittman, J.S., and Regehr, W.G. (1998). Calcium dependence and recovery kinetics of presynaptic depression at the climbing fiber to Purkinje cell synapse. J Neurosci *18*, 6147-6162.

Hallermann, S., Heckmann, M., and Kittel, R.J. (2010). Mechanisms of short-term plasticity at neuromuscular active zones of Drosophila. HFSP J *4*, 72-84.

Hamori, J., and Somogyi, J. (1983). Differentiation of cerebellar mossy fiber synapses in the rat: a quantitative electron microscope study. J Comp Neurol *220*, 365-377.

Hosoi, N., Sakaba, T., and Neher, E. (2007). Quantitative analysis of calcium-dependent vesicle recruitment and its functional role at the calyx of Held synapse. J Neurosci *27*, 14286-14298.

Magleby, K.L. (1987). Short-term changes in synaptic efficacy. In Synaptic function, G.M. Edelman, W.E. Gall, and W.M. Cowan, eds. (New York: John Wiley & Son), pp. 21-56.

Markram, H., Wang, Y., and Tsodyks, M. (1998). Differential signaling via the same axon of neocortical pyramidal neurons. Proc Natl Acad Sci U S A *95*, 5323-5328.

Mukherjee, K., Yang, X., Gerber, S.H., Kwon, H.B., Ho, A., Castillo, P.E., Liu, X., and Südhof, T.C. (2010). Piccolo and bassoon maintain synaptic vesicle clustering without directly participating in vesicle exocytosis. Proc Natl Acad Sci U S A *107*, 6504-6509.

Press, W.H., Teukolsky, S.A., Vetterling, W.T., and Flannery, B.P. (2002). Numerical recipes in C++: the art of scientific computing, 2nd edn (Cambridge, UK: Cambridge UP).

Sakaba, T., and Neher, E. (2001). Calmodulin mediates rapid recruitment of fast-releasing synaptic vesicles at a calyx-type synapse. Neuron *32*, 1119-1131.

Sargent, P.B., Saviane, C., Nielsen, T.A., DiGregorio, D.A., and Silver, R.A. (2005). Rapid vesicular release, quantal variability, and spillover contribute to the precision and reliability of transmission at a glomerular synapse. J Neurosci *25*, 8173-8187.

Saviane, C., and Silver, R.A. (2006a). Errors in the estimation of the variance: implications for multiple-probability fluctuation analysis. J Neurosci Methods *153*, 250-260.

Saviane, C., and Silver, R.A. (2006b). Fast vesicle reloading and a large pool sustain high bandwidth transmission at a central synapse. Nature *439*, 983-987.

Scheuss, V., Schneggenburger, R., and Neher, E. (2002). Separation of presynaptic and postsynaptic contributions to depression by covariance analysis of successive EPSCs at the calyx of Held synapse. J Neurosci *22*, 728-739.

Silver, R.A. (2003). Estimation of nonuniform quantal parameters with multiple-probability fluctuation analysis: theory, application and limitations. J Neurosci Methods *130*, 127-141.

Silver, R.A., Cull-Candy, S.G., and Takahashi, T. (1996). Non-NMDA glutamate receptor occupancy and open probability at a rat cerebellar synapse with single and multiple release sites. J Physiol *494*, 231-250.

Thomsen, L.B., Jorntell, H., and Midtgaard, J. (2010). Presynaptic calcium signalling in cerebellar mossy fibres. Front Neural Circuits *4*, 1.

Trommershäuser, J., Schneggenburger, R., Zippelius, A., and Neher, E. (2003). Heterogeneous presynaptic release probabilities: functional relevance for short-term plasticity. Biophys J *84*, 1563-1579.

Weis, S., Schneggenburger, R., and Neher, E. (1999). Properties of a model of Ca^{++}-dependent vesicle pool dynamics and short term synaptic depression. Biophys J *77*, 2418-2429.

Xu-Friedman, M.A., and Regehr, W.G. (2003). Ultrastructural contributions to desensitization at cerebellar mossy fiber to granule cell synapses. J Neurosci *23*, 2182-2192.

Young, S.M., Jr., and Neher, E. (2009). Synaptotagmin has an essential function in synaptic vesicle positioning for synchronous release in addition to its role as a calcium sensor. Neuron *63*, 482-496.
